# Identification of physical activity and sedentary behaviour dimensions that predict mortality risk in older adults: Development of a machine learning model in the Whitehall II accelerometer sub-study and external validation in the CoLaus study

**DOI:** 10.1016/j.eclinm.2022.101773

**Published:** 2022-12-13

**Authors:** Mathilde Chen, Benjamin Landré, Pedro Marques-Vidal, Vincent T. van Hees, April C.E. van Gennip, Mikaela Bloomberg, Manasa S. Yerramalla, Mohamed Amine Benadjaoud, Séverine Sabia

**Affiliations:** a Université Paris Cité, Inserm U1153, CRESS, Epidemiology of Ageing and Neurodegenerative Diseases, 10 Avenue de Verdun, 75010, Paris, France; b Department of Medicine, Internal Medicine, Lausanne University Hospital and University of Lausanne, Switzerland; c Accelting, Almere, the Netherlands; d Department of Internal Medicine, Maastricht University Medical Centre, the Netherlands; e School for Cardiovascular Diseases CARIM, Maastricht University, the Netherlands; f Department of Epidemiology and Public Health, University College London, UK; g Institute for Radiological Protection and Nuclear Safety (IRSN), Fontenay-Aux-Roses, France

**Keywords:** Physical activity, Mortality, Accelerometer, Prediction, Older adults

## Abstract

**Background:**

Identification of new physical activity (PA) and sedentary behaviour (SB) features relevant for health at older age is important to diversify PA targets in guidelines, as older adults rarely adhere to current recommendations focusing on total duration. We aimed to identify accelerometer-derived dimensions of movement behaviours that predict mortality risk in older populations.

**Methods:**

We used data on 21 accelerometer-derived features of daily movement behaviours in 3991 participants of the UK-based Whitehall II accelerometer sub-study (25.8% women, 60–83 years, follow-up: 2012–2013 to 2021, mean = 8.3 years). A machine-learning procedure was used to identify core PA and SB features predicting mortality risk and derive a composite score. We estimated the added predictive value of the score compared to traditional sociodemographic, behavioural, and health-related risk factors. External validation in the Switzerland-based CoLaus study (N = 1329, 56.7% women, 60–86 years, follow-up: 2014–2017 to 2021, mean = 3.8 years) was conducted.

**Findings:**

In total, 11 features related to overall activity level, intensity distribution, bouts duration, frequency, and total duration of PA and SB, were identified as predictors of mortality in older adults and included in a composite score. Both in the derivation and validation cohorts, the score was associated with mortality (hazard ratio = 1.10 (95% confidence interval = 1.05–1.15) and 1.18 (1.10–1.26), respectively) and improved the predictive value of a model including traditional risk factors (increase in C-index = 0.007 (0.002–0.014) and 0.029 (0.002–0.055), respectively).

**Interpretation:**

The identified accelerometer-derived PA and SB features, beyond the currently recommended total duration, might be useful for screening of older adults at higher mortality risk and for diversifying PA and SB targets in older populations whose adherence to current guidelines is low.

**Funding:**

National Institute on Aging; UK Medical Research Council; British Heart Foundation; Wellcome Trust; French National Research Agency; GlaxoSmithKline; Lausanne Faculty of Biology and Medicine; Swiss National Science Foundation.

**Research in context**

**Evidence before this study**

We searched PubMed for studies aiming at the identification of physical activity and sedentary behaviour features predicting mortality risk in older adults indexed in MEDLINE from January 1, 2000 to September 19, 2022, written in English or French, using the following search terms: ((“mortality” OR “death”) AND (“physical activity” OR “exercise” OR “sedentary” OR “accelerometry”) AND (predict∗) AND (“aged” OR “older”)). In the four identified relevant studies, accelerometer-derived features were reported as strong predictors of old-age mortality, however there was no clear consensus on their relative importance due to differing methodologies and a limited consideration of the multidimensional nature of movement behaviours. In addition, despite considerable heterogeneity in the older adult population, previous studies have neither examined the internal validity of their prediction models in subgroups, nor the external validity, precluding generalisation of results beyond the study population.

**Added value of this study**

Using a machine learning approach adapted to correlated predictors, we identified 11 features related to overall activity level, intensity distribution, and total duration, bouts duration, and frequency of physical activity and sedentary behaviour bouts as relevant predictors of 8-year mortality in a cohort of UK-based older adults. Composite score derived from these features showed good predictive ability in subgroups defined by age, sex, body mass index, and morbidity status. In addition, findings were validated in an independent Switzerland-based cohort.

**Implications of all the available evidence**

Adherence to current physical activity guidelines focussing mainly on the total duration in moderate-to-vigorous physical activity is low among older adults. The present study identified several dimensions of movement behaviours, in addition to total duration in physical activity, that predict mortality risk in older adults, above and beyond traditional risk factors. It might be relevant to integrate these dimensions into future physical activity guidelines.

## Introduction

Physical activity (PA) is an important determinant of healthy aging,^1^ with substantial evidence showing its protective role against morbidity and death.^2^ Emerging evidence also suggests that sedentary behaviour (SB) might be deleterious for health,^3^ leading to recent guidelines promoting reduction of time in SB in addition to practise of moderate to vigorous physical activity (MVPA).^4^, ^5^, ^6^ However, most of the evidence is primarily based on self-reported measures of movement behaviours.^7^ Although informative, these approaches cannot capture all dimensions of PA and SB, such as overall activity level, total and bouts duration, frequency, timing, and intensity distribution throughout the day.^8^ Accelerometers allow the measurement of multiple features that provide an in-depth characterisation of these dimensions. Furthermore, adherence to current PA and SB guidelines among older adults is relatively low^9^^,^^10^ with evidence accumulating that PA messages focusing on the total duration of PA may be perceived as unreachable goals and less efficient than tailored approaches.^11^^,^^12^ Identifying additional dimensions of PA and SB, collectively referred to as movement behaviours, relevant for health might benefit the population if they were perceived as easier to adhere to than increased time in MVPA.

To date, the few prospective studies that have evaluated the effectiveness and added value of objectively-assessed PA and SB features in predicting mortality among older adults suggest that some of these features (e.g. activity volume and fragmentation—the propensity to interrupt an activity bout) are among the strongest predictors of all-cause mortality.^13^, ^14^, ^15^, ^16^ However, these studies focused only on a limited set of PA and SB features, either investigated separately and/or without considering their correlated structure. Furthermore, whereas the older adult population is known to be heterogenous, neither internal validation in different subgroups of age, sex, or health condition nor external validation in an independent cohort has been conducted, limiting the generalisability of existing findings.

To address these limitations, the objective of this study was to identify PA and SB features that are core predictors of mortality risk among 21 accelerometer-assessed features in a large cohort of older adults. The added predictive value of the selected PA and SB features as compared to other known traditional risk factors was evaluated both in subgroups defined by age, sex, body mass index (BMI), or morbidity status, as well as in an independent external cohort.

## Methods

### Study participants

The Whitehall II study is an ongoing prospective cohort study established in 1985–1988 among 10,308 British civil servants^17^ with clinical examinations every four–five years since inception. An accelerometer measure was added to the 2012–2013 wave of data collection for participants seen at the London clinic and those living in the south-eastern regions of England who underwent clinical examination at home. This study followed the reporting guidelines for prediction model and development (TRIPOD).

### PA and SB features

Participants were requested to wear a tri-axial accelerometer (GENEActiv Original; Activinsights Ltd, Kimbolton, UK) on their non-dominant wrist for nine consecutive 24-h days. Accelerometer data, sampled at 85.7 Hz and expressed relative to gravity (1 *g* = 9.81 m/s^2^), were processed using GGIR R package (version 2.3-3).^18^ Euclidean Norm of raw accelerations Minus One with negative numbers rounded to zero was calculated^19^ and corrected for calibration error and non-wear time.^18^ Sleep periods were detected using a validated algorithm guided by a sleep log.^20^ Average acceleration over 60-s epochs <40 milligravity (m*g*) during waking period was classified as SB, 40–99 m*g* as light-intensity PA (LIPA), and ≥100 m*g* as MVPA.^18^^,^^21^

We searched the most commonly used movement behaviour features in the literature that could be extracted from accelerometer data using the GGIR R package and identified 21 features covering six *dimensions* of daily movement behaviours. *Overall activity level* was measured using average acceleration. *Total duration* was investigated using total time in SB, LIPA, and MVPA. *Bouts duration* was measured using mean duration of sedentary/LIPA/MVPA bout (i.e. uninterrupted episode), time accumulated in sedentary bouts lasting <10 min, 10–30 min, or ≥30 min, and time accumulated in LIPA/MVPA bouts lasting < or ≥10 min. *Frequency* was characterized by the number of sedentary/LIPA/MVPA bouts and the number of days with ≥30 min of MVPA.^22^ *Timing of PA* was defined as the start of the most active 5 consecutive hours during the waking period. *Intensity distribution* was characterized by the intercept and the gradient of the linear relationship between intensity and time accumulated in that intensity.^21^ More information on these features is provided in Supplementary Table S1.

### Mortality

Deaths from any cause were ascertained using mortality records drawn from the UK Office for National Statistics Mortality Register until 28th February 2021. The tracing exercise was carried out using the unique National Health Service identification number of each participant. The start of follow-up was the screening date at the 2012–2013 study wave, and participants were censored at the date of death or end of the follow-up, whichever came first.

### Covariates

Covariates were selected to represent traditional risk factors for mortality and were drawn from questionnaires and clinical evaluations conducted in 2012–2013, as well as from electronic health records. They included: *socio-demographic* factors (age, sex, ethnicity, marital status, and education level), *behavioural* (smoking status, alcohol consumption, and fruit and vegetable consumption) and *health-related* factors (BMI, hypertension, hyperlipidemia, diabetes, number of limited basic activities of daily living (ADL), number of limited instrumental ADL (IADL), and number of chronic diseases among coronary heart disease, stroke, heart failure, arthritis, cancer, depression, dementia, Parkinson's disease, and chronic obstructive pulmonary disease) (see Supplementary Methods 1 for details).

### Statistical analyses

Three sets of analyses were conducted and are described in following paragraphs: 1) development of PA and SB composite scores that predict mortality risk in the Whitehall accelerometer sub-study; 2) internal validation of the composite scores; and 3) external validation.

### Development of PA and SB composite scores that predict mortality risk

In exploratory analysis we first examined the association of each of the 21 PA and SB features with mortality, in separate models, using a Cox regression model adjusted for traditional risk factors (socio-demographic, behavioural, and health-related factors). Then, we identified among these 21 features the ones most relevant for mortality prediction using sparse Partial Least Square regression for censored data, a machine learning method adapted to highly correlated features.^23^^,^^24^ This method jointly performs predictor selection and dimension reduction by deriving composite scores composed of the most relevant features, which are tuned by cross-validation (Supplementary Methods 2). The two composite scores resulting from this analysis, referred to as composite score 1 and composite score 2, represented the best set of accelerometer-derived predictors of all-cause mortality.

### Internal validation

The association between the composite scores and mortality was examined using hazard ratio (HR) derived from Cox regression. First, Model 1 included only traditional risk factors. Then, in Model 2, composite score 1 was added to Model 1 and finally, in Model 3, composite score 2 was further included in Model 2. Potential non-linear associations of composite scores with mortality were tested using likelihood ratio test comparing models with only a linear term against models with cubic spline terms. As no evidence of a non-linear relationship of composite scores with mortality was found (p = 0.82 and 0.23, respectively for composite scores 1 and 2), composite scores were entered as linear terms in all analyses.

Predictive accuracy was assessed using: Royston's modified R^2^, a measure of the overall prediction performance of the model; Akaike information criterion (AIC), an estimator of a model's prediction error considering its complexity; sensitivity and specificity as measures of classification accuracy (the optimal cut-off was established by maximizing the Youden index); and Harrell's C-statistic for survival models to measure discrimination. With the exception of AIC, higher values indicate better model performance. To evaluate the added predictive performance of composite scores 1 and 2 compared to traditional risk factors, the AIC and C-statistic of Models 2 and 3 were compared with Model 1 as the reference. Additionally, Model 3 was compared with Model 2 as reference to estimate the gain in predictive performance of composite score 2 as compared to composite score 1. Associated confidence intervals were computed using 1000 bootstrap replications. In the absence of an association with mortality in the fully-adjusted model (Model 3) and performance gain of composite score 2 compared to composite score 1, further validation analyses were conducted using composite score 1 only.

In sensitivity analysis, we examined the potential impact of the duration of the waking period on our findings by repeating the analysis using PA and SB features standardized to the duration of the waking period (as described in Supplementary Table S1) before inclusion in the selection procedure.

Previous studies suggested that the association between movement behaviours and mortality may differ according to different risk factors, including age, sex, BMI, and morbidity status.^25^^,^^26^ Accordingly, we examined the association of composite score 1 with mortality risk as well as its added predictive value in the following subgroups: (i) age <74 and ≥74 years old (to allow enough mortality cases in each group); (ii) men and women, (iii) participants with normal BMI (<25 kg/m^2^), overweight (BMI 25–29.9 kg/m^2^), and obesity (BMI ≥30 kg/m^2^); and (iv) presence and absence of morbidity (defined as one or more chronic disease(s)).

### External validation

To assess generalisability, the association of the retained composite score 1 with mortality and its added predictive value were also evaluated in 1648 participants aged ≥60 years from the CoLaus accelerometer sub-study (Lausanne, Switzerland).^27^ Socio-demographic, behavioural, health-related, and accelerometer variables were extracted from questionnaires and clinical evaluations at the 2014–2017 study wave (see details in Supplementary Methods 3).

At each wave, Whitehall II participants provided informed written consent. All participants from the CoLaus study gave their signed informed consent before entering the study. Research ethics approval was obtained from the National Health Service London—Harrow Research Ethic Committee (latest reference number 85/0938) for the Whitehall II study and from the Ethics Commission of Canton Vaud for CoLaus study (239/09, decision of 21st June 2021).

All analyses were undertaken using R version 4.1.2 (http://www.r-project.org) with a two-sided p < 0.05 considered statistically significant. All relevant R scripts and documentation can be accessed via the project repository (https://github.com/MathildeChen/PA-SB-dimensions-mortality-Whitehall). Technical information regarding raw accelerometer data processing can be found in the GGIR repository (https://github.com/wadpac/GGIR).

### Role of the funding source

The funders of the study had no role in study design, data collection, data analysis, data interpretation, or writing of the report. The first author had full access to the datasets and all authors approved the final manuscript for submission.

## Results

Among the 6308 participants in the 2012–2013 wave of data collection in Whitehall II, 4880 were invited to participate in the accelerometer sub-study, of whom 4008 had valid accelerometer data (Supplementary Figure S1). Excluding those with missing covariates led to an analytical sample of 3991 (mean age: 69.4 years, 25.8% women). Over a mean follow-up of 8.1 (standard deviation [SD] = 1.3) years, 410 deaths were recorded. Deceased participants were more likely to be older, married/cohabitating, less educated, have a worse cardiovascular risk profile, more comorbidities, and more functional limitations compared to those who survived over the follow-up (Table 1). Participants who died spent more time in SB, which was accumulated in longer, uninterrupted episodes, and less time in higher activity intensities compared to surviving participants. Later timing of PA was observed among surviving participants (Table 2). Differences for each of these features were also evident in separate analyses adjusted for socio-demographic, behavioural, and health-related factors with the exception of mean duration of LIPA bouts (Hazard Ratio (HR) for 1-SD increment [95% confidence interval (CI)]: 0.94 [0.86, 1.04], p = 0.22), time in ≥10 min LIPA bouts (HR [95% CI]: 0.93 [0.84, 1.03], p = 0.16), and timing of PA (HR [95% CI]: 0.96 (0.87, 1.06), p = 0.41) (Table 2). Most of accelerometer-derived features exhibited strong correlation, with the exception of timing of PA (absolute coefficient ≤ 0.05) (Supplementary Figure S2), leading us not to mutually adjust models for all features.

**Table 1:** Sample characteristics in 2012–2013 by mortality status at the end of the follow-up (February 2021) in the Whitehall II accelerometer sub-study.

	All-cause mortality		p-value
	No (N = 3581)	Yes (N = 410)	
**Socio-demographic factors**			
Age (years), M (SD)	68.9 (5.5)	73.8 (5.3)	<0.001
Women	938 (26.2%)	92 (22.4%)	0.10
Non-white ethnicity	259 (7.2%)	36 (8.8%)	0.26
Education			
No academic qualifications	321 (9.0%)	49 (12.0%)	<0.001
Lower secondary school	1128 (31.5%)	144 (35.1%)	
Higher secondary school	984 (27.5%)	127 (31.0%)	
University	843 (23.5%)	72 (17.6%)	
Higher degree	305 (8.5%)	18 (4.4%)	
Not married/cohabitating	873 (24.4%)	137 (33.4%)	<0.001
**Behavioural factors**			
Smoking status			
Never smoker	1787 (49.9%)	174 (42.4%)	0.017
Past smoker	1599 (44.7%)	210 (51.2%)	
Current smoker	195 (5.4%)	26 (6.3%)	
Alcohol intake			
0 unit/week	702 (19.6%)	110 (26.8%)	0.003
1–14 units/week	2039 (56.9%)	215 (52.4%)	
>14 units/week	840 (23.5%)	85 (20.7%)	
Fruit and vegetable intake			
Less than daily	718 (20.1%)	108 (26.3%)	0.001
Daily	752 (21.0%)	100 (24.4%)	
Twice daily or more	2111 (59.0%)	202 (49.3%)	
**Health-related factors**			
Body mass index			
Normal (<25 kg/m^2^)	1383 (38.6%)	165 (40.2%)	0.80
Overweight (25–29.9 kg/m^2^)	1549 (43.3%)	171 (41.7%)	
Obesity (≥30 kg/m^2^)	649 (18.1%)	74 (18.0%)	
Hypertension	1804 (50.4%)	262 (63.9%)	<0.001
Hyperlipidemia	1792 (50.0%)	229 (55.9%)	0.026
Diabetes	434 (12.1%)	80 (19.5%)	<0.001
Number of chronic diseases,^a^ M (SD)	0.51 (0.72)	0.84 (0.93)	<0.001
Number of basic ADL,^b^ M (SD)	0.15 (0.59)	0.47 (1.13)	<0.001
Number of IADL,^c^ M (SD)	0.18 (0.58)	0.40 (0.90)	<0.001

Data are N (%), otherwise specified.

Abbreviations: SD: standard deviation; ADL: activities of daily living; IADL: instrumental activities of daily living.

a Chronic disease include coronary heart disease, stroke, heart failure, arthritis, cancer, depression, dementia, Parkinson's disease, and chronic obstructive pulmonary disease.

b Basic ADL include difficulty in dressing, walking, bathing, eating, getting in bed, and using the toilet.

c IADL include difficulty in cooking, shopping for groceries, making telephone calls, taking medication, doing housework, and managing money.

**Table 2:** Features of physical activity and sedentary behaviour in 2012–2013 by mortality status at the end of the follow-up (February 2021) and their separate association with mortality in the Whitehall II accelerometer sub-study.

	Overall (N = 3991)	All-cause mortality		p-value^a^	Association with mortality^b^	
		No (N = 3581)	Yes (N = 410)		HR (95% CI)	p-value
**Overall activity level**						
Mean acceleration (m*g*)	31.8 (9.7)	32.3 (9.7)	27.2 (8.4)	<0.001	0.76 (0.67, 0.87)	<0.001
**Total duration (min/day)**						
SB	717.9 (100.1)	713.0 (98.5)	760.5 (104.6)	<0.001	1.22 (1.10, 1.35)	<0.001
LIPA	210.3 (69.1)	213.0 (67.6)	186.9 (77.0)	<0.001	0.86 (0.78, 0.95)	0.0027
MVPA	56.1 (38.5)	58.2 (38.6)	37.3 (33.0)	<0.001	0.74 (0.64, 0.85)	<0.001
**Bouts duration**						
Mean duration (min) of activity bout of						
SB	11.5 (5.9)	11.1 (5.2)	14.3 (9.8)	<0.001	1.07 (1.02, 1.14)	0.011
LIPA	2.4 (0.4)	2.4 (0.4)	2.4 (0.4)	0.118	0.94 (0.86, 1.04)	0.22
MVPA	2.3 (0.9)	2.3 (0.9)	2.0 (1.1)	<0.001	0.87 (0.77, 0.99)	0.03
Time (min/day) in <10 min bouts of						
SB	147.1 (41.0)	148.6 (40.1)	134.6 (45.8)	<0.001	0.89 (0.81, 0.98)	0.023
LIPA	184.0 (54.1)	186.4 (52.7)	162.9 (61.2)	<0.001	0.85 (0.77, 0.93)	0.001
MVPA	43.4 (27.6)	45.0 (27.5)	30.0 (24.7)	<0.001	0.75 (0.65, 0.86)	<0.001
Time (min/day) in ≥10 min bouts of						
LIPA	26.4 (21.3)	26.6 (21.2)	24.0 (21.6)	0.02	0.93 (0.84, 1.03)	0.16
MVPA	12.7 (17.2)	13.3 (17.5)	7.3 (12.5)	<0.001	0.84 (0.72, 0.98)	0.023
Time (min/day) in 10–29.9 min bouts of						
SB	185.7 (45.3)	187.4 (44.4)	171.0 (50.1)	<0.001	0.90 (0.82, 0.99)	0.031
Time (min/day) in ≥30 min bouts of						
SB	385.0 (143.0)	377.0 (138.3)	454.9 (163.8)	<0.001	1.22 (1.11, 1.34)	<0.001
**Frequency**						
Number (N/day) of bouts of						
SB	71.8 (16.0)	72.5 (15.5)	66.2 (18.6)	<0.001	0.87 (0.80, 0.96)	0.005
LIPA	86.0 (21.6)	87.1 (21.0)	76.3 (24.4)	<0.001	0.83 (0.75, 0.92)	<0.001
MVPA	23.2 (13.6)	24.0 (13.4)	16.6 (12.7)	<0.001	0.76 (0.67, 0.87)	<0.001
Number of days with ≥30 min of MVPA	4.5 (2.5)	4.7 (2.4)	3.1 (2.8)	<0.001	0.79 (0.71, 0.88)	<0.001
**Intensity distribution**						
Intensity intercept	12.4 (0.7)	12.3 (0.6)	12.7 (0.7)	<0.001	1.20 (1.08, 1.34)	<0.001
Intensity gradient	−2.08 (0.22)	−2.07 (0.21)	−2.20 (0.25)	<0.001	0.82 (0.74, 0.91)	<0.001
**Timing**^c^						
Timing of PA (hours)	10 h 12 min (1 h 36 min)	10 h 12 min (1 h 36 min)	10 h 0 min (1 h 48 min)	0.01	0.96 (0.87, 1.06)	0.41

Data are mean (standard deviation).

Abbreviations: HR: hazard ratio; CI: confidence interval; SB: sedentary behaviour; LIPA: light-intensity physical activity; MVPA: moderate-to-vigorous physical activity; PA: physical activity.

a Estimated using Student's t-test.

b Associations of PA and SB features with mortality risk (HR for one standard deviation increment) were examined in separate models adjusted for sociodemographic, behavioural, health-related factors.

c Timing of five most active hours during the waking period.

### Identified PA and SB features

**Fig. 1:**
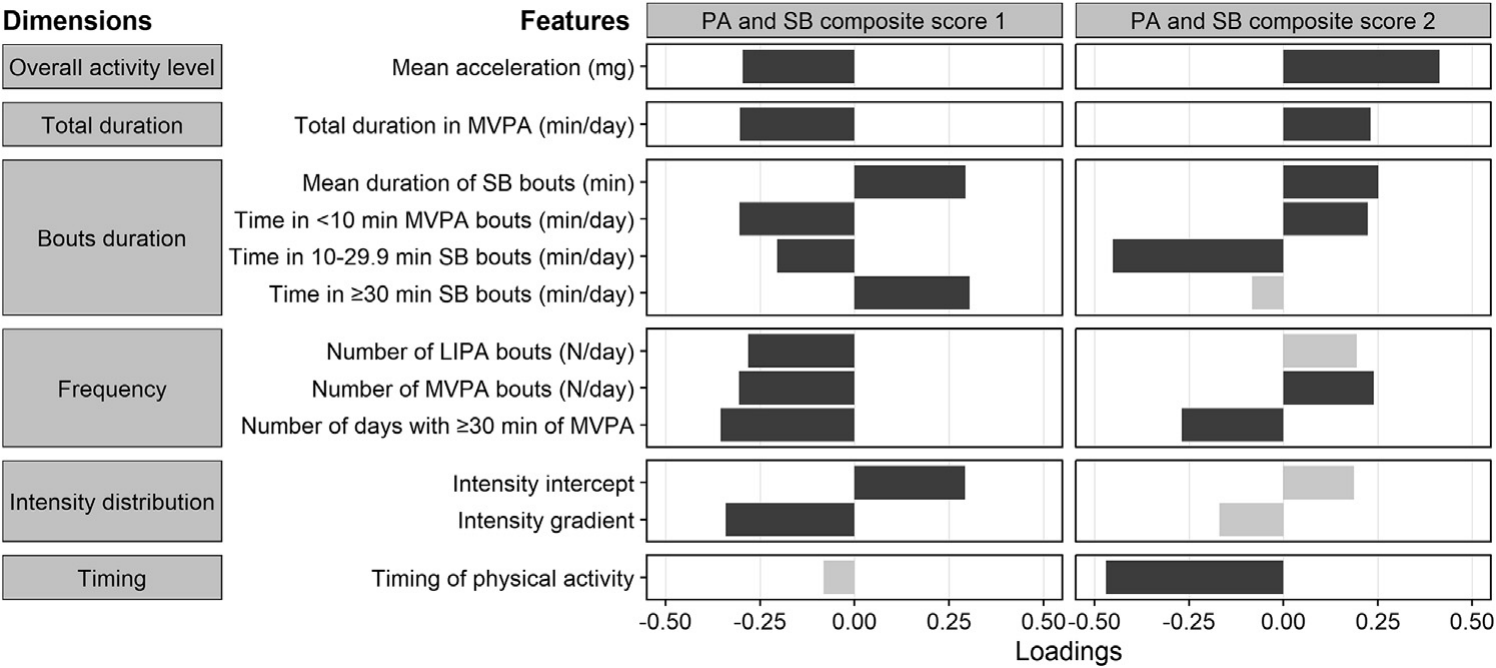
**Factor loadings of physical activity and sedentary behaviour variables in composite scores identified as predictors of mortality risk**. PA: physical activity; SB: sedentary behaviour; MVPA: moderate-to-vigorous physical activity; LIPA: light-intensity physical activity. Dark grey: factor loading absolute value ≥0.2; light grey: factor loading absolute value <0.2.

Of the 21 PA and SB features investigated, 12 related to overall activity, total duration, bouts duration, frequency, intensity distribution, and timing of PA were selected and computed as two composite scores by the machine learning procedure (Fig. 1). The selected features substantially contributed to composite score 1 with similar magnitude (absolute loads from 0.20 to 0.35), with the exception of timing of PA (−0.08). Higher composite score 1 represented lower overall activity level, less time in MVPA, accumulation of SB time in longer bouts, less frequent bouts of LIPA and MVPA, less days complying the recommended daily practise of 30 min of MVPA, more time in low intensity activity (reflected by higher intensity distribution intercept), and less well distributed activities of different intensities. In contrast, timing of PA contributed the most to composite score 2, followed by time in sedentary bouts of 10–30 min and average acceleration. In sensitivity analysis using PA and SB features standardized according to waking period duration, a two composite scores solution was also identified, although features related to overall activity level and timing of PA dimensions were not selected (Supplementary Figure S3).

### Internal validation

**Fig. 2:**
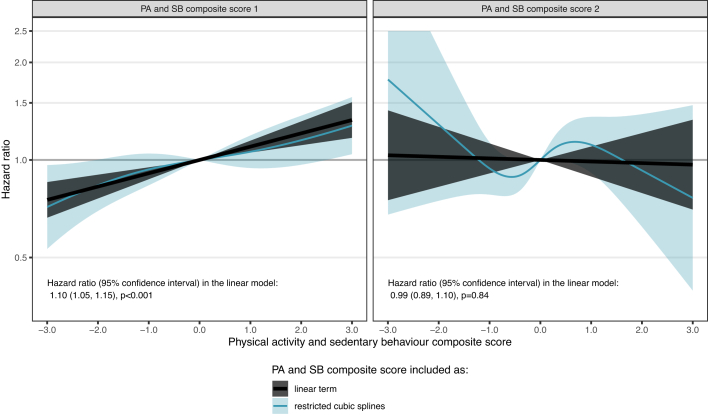
**Association of the physical activity and sedentary behaviour composite scores with mortality in Whitehall II accelerometer sub-study**. Estimated using a model including sociodemographic, behavioural, health-related factors, and both composite scores 1 and 2. Shaded black and blue areas around estimates based on linear term and restricted cubic splines represent 95% confidence intervals.

In a model including traditional risk factors (sociodemographic, behavioural, and health-related factors) and composite scores 1 and 2, a 1-SD increase in composite score 1 was associated with a 10% increase in mortality risk (HR [95% CI]: 1.10 [1.05, 1.15], p < 0.001), while no association was found for composite score 2 (HR [95% CI]: 0.99 [0.89, 1.10], p = 0.84) (Fig. 2 and Table 3). Compared to a model including traditional risk factors (Model 1), a model additionally adjusted for composite score 1 (Model 2) had better goodness of fit in terms of R^2^ and AIC (ΔAIC [95% CI]: −19.3 [−45.1, −5.6]), better classification accuracy in terms of sensitivity and specificity, and had a significantly higher C-index (ΔC-statistic [95% CI]: 0.007 [0.002, 0.014]) as shown in Table 3. When adding composite score 2 (Model 3) to this model (Model 2), a lower sensitivity but greater specificity was found, but the predictive performance was similar in terms of AIC and C-statistics (Table 3). In absence of association between composite score 2 and mortality and of performance gain from a model adding composite score 2 to one including composite score 1, further analyses were conducted using composite score 1 only. Similar results were observed in sensitivity analysis using composite scores based on standardised PA and SB features on waking period duration both for comparison of Model 2 to Model 1 (ΔAIC [95% CI] and ΔC-statistic [95% CI]: −22.0 [−49.4, −7.6] and 0.007 [0.002, 0.015], respectively) and of Model 3 to Model 2 (ΔAIC [95% CI] and ΔC-statistic [95% CI]: 2.0 [−8.4, 2.0] and −0.00001 [−0.0003, 0.004], respectively) (Supplementary Table S2).

**Table 3:** Predictive performance of physical activity and sedentary behaviour composite scores 1 and 2 for mortality risk in the Whitehall II accelerometer sub-study (N cases/N total = 410/3991, mean [standard deviation] follow-up = 8.1 [1.3] years).

	HR (95% CI) PA and SB composite scores	Royston's R^2^ (95% CI)	AIC (95% CI)	ΔAIC (95% CI)	ΔAIC (95% CI)	Sensitivity % (95% CI)^a^	Specificity % (95% CI)^a^	C-index (95% CI)	ΔC-index (95% CI)	ΔC-index (95% CI)
Model 1 b	NA	0.366 (0.328, 0.424)	6374.6 (5801.3, 6989.1)	*Ref.*	NA	69.9 (56.4, 87.9)	66.4 (47.1, 82.3)	0.751 (0.731, 0.778)	*Ref.*	NA
Model 2 c	PA and SB score 1: 1.10 (1.06, 1.14), p < 0.001	0.382 (0.344, 0.439)	6355.3 (5794.5, 6960.7)	−19.3 (−45.1, −5.6)^e^	*Ref.*	70.3 (53.2, 85.2)	67.1 (51.4, 83.7)	0.758 (0.738, 0.784)	0.007 (0.002, 0.014)^e^	*Ref.*
Model 3 d	PA and SB score 1: 1.10 (1.05, 1.15), p < 0.001	0.381 (0.345, 0.439)	6357.2 (5798.4, 6963.5)	−17.4 (−44.5, −4.3)^e^	2.0 (−7.5, 1.9)	67.2 (50.5, 86.1)	70.4 (52.9, 86.1)	0.758 (0.738, 0.784)	0.007 (0.003, 0.014)^e^	−0.0001 (−0.0003, 0.0041)
	PA and SB score 2: 0.99 (0.89, 1.10), p = 0.84									

Abbreviations: CI: confidence interval; AIC: Akaike information criterion; C-index: Harrell's C-index.

a Youden index cutoff points for the calculation of the sensitivity and specificity: 0.363 (95% CI: [0.301, 0.475]) for model 1, 0.374 (95% CI: [0.318, 0.489]) for model 2, and 0.381 (95% CI: [0.354, 0.424]) for Model 3.

b Model 1: Model includes socio-demographic, behavioural, and health-related factors.

c Model 2: Model 1 additionally adjusted for the physical activity and sedentary behaviour composite score 1.

d Model 3: Model 2 additionally adjusted for the physical activity and sedentary behaviour composite score 2.

e Confidence intervals (calculated based on 1000 bootstrap samples) not containing zero indicate significant differences in predictive performance between Model 1 and Model 2, and between Model 2 and Model 3.

**Fig. 3:**
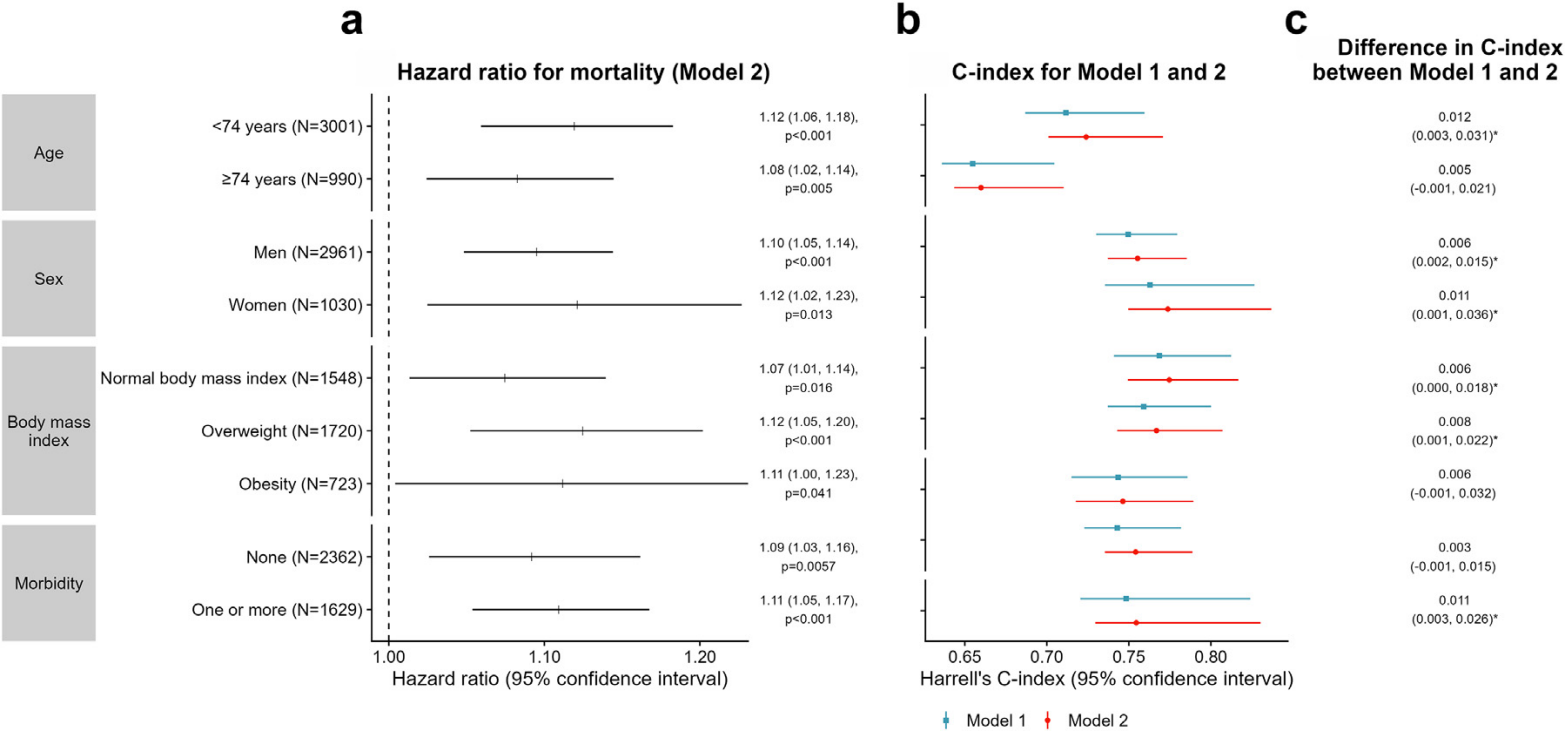
**Association of the physical activity and sedentary behaviour composite score 1 with mortality risk and its predictive performance by subgroups defined by age, sex, body mass index, and morbidity status in Whitehall II accelerometer sub-study**. C-index: Harrell's C-index. Model 1: Model includes socio-demographic, behavioural, and health-related factors. Model 2: Model 1 additionally adjusted for the physical activity and sedentary behaviour composite score 1. ∗ Confidence intervals not containing zero indicate significant difference in predictive performance between Model 1 and Model 2.

The association of composite score 1 with mortality and its added predictive value were examined in subgroups defined by age (<74 and ≥74 years), sex, BMI (normal, overweight, and obesity), and morbidity status (subgroup characteristics shown in Supplementary Table S3). A significant association between composite score 1 and mortality was observed in all subgroups (Fig. 3). A 1-SD increase in the composite score was associated with a higher mortality risk ranging from 8% among those aged ≥74 years (HR [95% CI]: 1.08 [1.02, 1.14], p = 0.005) to 12% in participants aged <74 years old (1.12 [1.06, 1.18], p < 0.001), women (1.12 [1.02, 1.23], p = 0.013), and overweight participants (1.12 [1.05, 1.20], p < 0.001). The association was marginally significant among participants with obesity (1.11 [1.00, 1.23], p = 0.041).

Compared to a model adjusted for traditional risk factors (Model 1), including composite score 1 improved the predictive accuracy in terms of C-index among all sub-groups although the improvement did not reach significance for all groups (Fig. 3). Significant improvement in predictive performance for mortality was observed among participants <74 years (ΔC-index [95% CI]: 0.012 [0.003, 0.031]), men (0.006 [0.002, 0.015]), women (0.011 [0.001, 0.036]), participants with normal BMI (0.006 [0.000, 0.018]), overweight participants (0.008 [0.001, 0.022]), and participants with at least one chronic disease (0.011 [0.003, 0.026]). A similar trend was observed among those aged ≥74 and participants with obesity, although no significant difference in C-index was found for these groups. Results were consistent when considering the AIC of the models (Supplementary Tables S4–S7) instead of C-index, except for women (Supplementary Table S5) and participants with normal BMI (Supplementary Table S6) where the CI of the ΔAIC included zero.

### External validation

A total of 1329 participants aged ≥60 years (mean age: 69.5 years, 56.7% women) of the CoLaus accelerometer sub-study composed the validation cohort (Supplementary Methods 3, Supplementary Figure S4, Supplementary Table S8), of whom 105 died after a mean (SD) follow-up of 3.8 (0.7) years. Compared to Whitehall II, participants of CoLaus were more likely to be women (25.8% in Whitehall II vs 56.7% in CoLaus), of white ethnicity (92.6 vs 95.0%), to live alone (25.3 vs 56.7%), smoke (5.5 vs 14.6%), have a lower alcohol intake (20.3 vs 24.5%) and higher consumption of fruit and vegetables (59.0 vs 69.2%). The proportion of participants with a high BMI (18.1 vs 22.1%) and hypertension (51.7 vs 55.1%) was higher in CoLaus, while hyperlipidemia (50.6 vs 38.0%), morbidity (40.8 vs 20.8%), and functional limitations (9.7 vs 8.5%) were more frequent in Whitehall II. In addition, participants included in CoLaus tended to have higher PA and SB levels (mean [SD] acceleration: 31.8 [9.7] m*g* in Whitehall vs 38.2 [11.8] mg in CoLaus), compared to participants from Whitehall II, which might partly reflect difference in wrist placement between the studies (dominant in CoLaus, non-dominant in Whitehall).

**Fig. 4:**
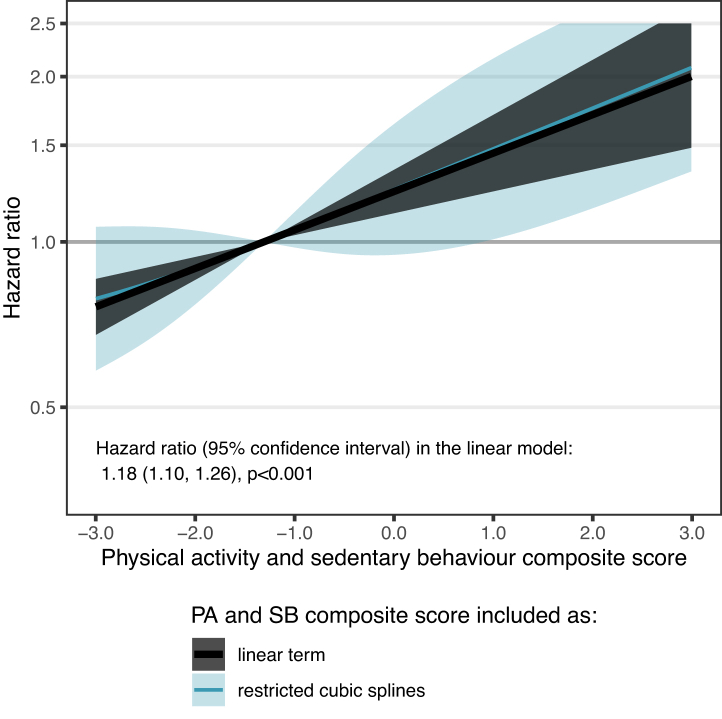
**Association of the physical activity and sedentary behaviour composite score 1 with mortality risk in the CoLaus study**. Estimated using a model including sociodemographic, behavioural, health-related factors, and composite score 1. Shaded black and blue areas around estimates based on linear term and restricted cubic splines represent 95% confidence intervals.

As in the Whitehall II, there was no evidence of a non-linear relationship of composite score 1 with mortality (p = 0.87). A 1-SD increase in composite score 1 was associated with a 18% higher mortality in this validation cohort (HR [95% CI]: 1.18 [1.10, 1.26], p < 0.001) (Fig. 4). Compared to the model including traditional risk factors, the model additionally adjusted for composite score 1 showed a significantly better predictive performance (ΔAIC [95% CI]: −17.8 [−41.8, −4.3]; ΔC-statistic [95% CI]: 0.029 [0.002, 0.055]; Table 4).

**Table 4:** Predictive performance of the physical activity and sedentary behaviour composite score 1 for mortality risk in CoLaus accelerometer sub-study (N cases/N total = 105/1329, mean [standard deviation] follow-up = 3.8 [0.7] years).

	HR (95% CI) PA and SB composite score 1	Royston's R^2^ (95% CI)	AIC (95% CI)	ΔAIC (95% CI)	Sensitivity % (95% CI)^a^	Specificity % (95% CI)^b^	C-index (95% CI)	ΔC-index (95% CI)
Model 1 c	NA	0.392 (0.349, 0.556)	1121.4 (876.0, 1308.8)	*Ref.*	56.1 (49.5, 90.3)	84.2 (50.8, 90.3)	0.757 (0.716, 0.830)	*Ref.*
Model 2 b	1.18 (1.10, 1.26), p < 0.001	0.430 (0.394, 0.593)	1103.6 (856.7, 1287.9)	−17.8 (−41.8, −4.3)^d^	74.2 (55.4, 88.8)	75.4 (56.4, 90.4)	0.785 (0.737, 0.856)	0.029 (0.002, 0.055)^d^

CI: confidence interval; AIC: Akaike information criterion; C-index: Harrell's C-index; PA, physical activity; SB, sedentary behaviour.

a Youden index cutoff points for the calculation of the sensitivity and specificity: 0.403 (95% CI: [0.345, 0.578]) for Model 1 and 0.496 (95% CI: [0.391, 0.628]) for Model 2.

b Model 2: Model 1 additionally adjusted for the physical activity and sedentary behaviour composite score 1.

c Model 1: Model includes socio-demographic, behavioural, and health-related factors.

d Confidence intervals (calculated based on 1000 bootstrap samples) not containing zero indicate significant differences in predictive performance between Model 1 and Model 2.

## Discussion

This study using accelerometer-derived measures of PA and SB features in older adults presents three key findings. One, overall activity level, bouts duration, frequency, and intensity distribution were identified as important dimensions of movement behaviours associated with risk of mortality in older adults, in addition to the currently-recommended total duration in MVPA. Two, the developed composite score comprising 11 modifiable PA and SB features predicted mortality risk above and beyond socio-demographic, behavioural, and health-related risk factors. Subgroup analysis suggested that the score was associated with mortality in subgroups defined by age, sex, BMI, and morbidity status; its added predictive value reached significance both in men and women, and in the younger group (<74 years), those presenting morbidity, and in groups without obesity. Three, the association of the PA and SB composite score with mortality and its added predictive value was confirmed in the external validation cohort.

To our knowledge, few studies have previously investigated the effectiveness and added value of the objectively-assessed features of PA and SB in predicting mortality in older adults.^13^, ^14^, ^15^, ^16^ One prospective study using data from The Rush Memory and Aging Project (N = 1249; mean age: 80 years), focused on features measured during specific gait tests such as Timed Up and Go test.^13^ Other studies examined daily life activities in participants aged ≥50 from the UK Biobank^14^ or the National Health And Nutrition Examination Survey.^15^^,^^16^ They included seven to 25 features covering up to 4 dimensions of movement behaviour and used variables selection methods that do not take into account the high correlation between these features. They identified several features to be among the strongest predictors of 5-year mortality in comparison with other traditional mortality predictors. Overall activity^14^^,^^15^ and active to sedentary transition probability,^15^^,^^16^ a marker of the fragmentation of activity bouts as mean duration of activity bouts in the present study, were selected in two different studies, but heterogeneity between studies in included features and in ranking methodologies makes further comparisons difficult. None of these studies examined the external validity of findings, as recommended for studies using a machine learning approach,^28^ limiting the generalisability of the findings.

We extend previous studies by developing and validating a PA and SB composite score for mortality risk using a machine learning approach adapted to correlated data that simultaneously performs variable selection and dimension reduction. Dimensions investigated in previous studies were included as well as two additional dimensions (frequency and intensity distribution). The composite score derived from 11 features distributed in five dimensions of movement behaviour was found to predict mortality above and beyond traditional sociodemographic, behavioural, and health-related risk factors both in the derivation and validation cohorts, indicating generalisability of findings. In addition to the currently recommended guidelines of longer time in MVPA across several days of the week,^4^, ^5^, ^6^ we identified higher frequency of LIPA and MVPA bouts, more fragmented SB, and a more uniform (higher gradient and lower intercept) intensity distribution to be associated with reduced risk of mortality in older adults. The magnitude of factors loadings of these features was similar as the one of total duration in MVPA, denoting their equal contribution. Contrary to the recommendation of reducing sedentary time found in recent guidelines,^4^, ^5^, ^6^ we did not find evidence that total time in SB was an important predictor of mortality, while the way it was accumulated appeared to be important.

The composite score developed in the present study was associated with mortality in all subgroups defined by sex, age, BMI, and morbidity status, although the degree to which it improved predictive accuracy compared to traditional risk factors varied between subgroups. The composite score did not improve mortality risk prediction in participants aged ≥74 years. We observed that this oldest old group had overall low levels of PA and high sedentarity with low variability, suggesting that limitations in the functional capacity itself may partially explain the reduced ability of the composite score to predict risk of mortality in line with a previous hypothesis.^29^ The added predictive value of the composite score was also uncertain for participants with obesity although the lower sample size for this group may explain lack of significance. There was no clear evidence of added value of the composite score in predicting mortality in participants without chronic diseases. The progression to death in these individuals located upstream of the morbidity-mortality process may be more complex and heterogeneous, and may involve a larger set of predictive factors, diminishing the added importance of any one of them compared to the whole. In contrast, the added predictive value of the composite score found among participants with chronic diseases highlights the importance of physical activity in monitoring the progression of chronic diseases and its rehabilitation.^30^^,^^31^

Our study has limitations. First, although we used data on a large set of PA and SB features, accelerometery measurement does not provide information on posture and context of PA and SB, including use of assisting device, precluding us from examining these two important dimensions of movement behaviour. Second, participants of the Whitehall II cohort were all employed at recruitment in 1985–1988, predominantly men, with better health than the general population. However, the use of external (CoLaus independent cohort) validation showed that these features have little influence on the robustness of our findings. Third, some of the covariates were self-reported (behavioural factors and limitations in ADL/IADL), their association with mortality may have been underestimated leading to potential residual confounding in the association between the composite score and mortality. Finally, the focus of this study was on identifying features of PA and SB during waking period important for health as current PA guidelines highlight this research need.^32^ However, there is emerging research including sleep as part of the 24-h movement behaviours. The way to examine sleep features along with PA and SB features during the waking period is still debated,^33^ particularly among older adults who tend to have more sleep interruptions, although independently from their individual choices. Identification of 24-h PA, SB, and sleep features that predict mortality requires further investigation.

Though there have been recent changes in PA guidelines as the measurement of movement behaviours has improved,^7^ adherence to current recommendations of increasing time in MVPA while reducing sedentary time among older adults remains poor.^9^^,^^10^ It is suggested that public health messages targeting total duration of PA or SB, such as 150 min/week of MVPA, are perceived as (1) unattainable, (2) far from the targets people are willing to achieved, and (3) embedded within negative representations linking PA with sport.^11^^,^^12^ The present study identified 10 PA and SB features, in addition to time in MVPA, associated with mortality risk that could be used as new additional levers of action in tailored interventions in a population that maintains low levels of activity. The precise design of these interventions is beyond the scope of this study and requires further investigations but, overall, diversification of physical activity targets, for example, through tools promoting breaks in sitting activities or personalized messages scheduled to incite people to go for a walk might be an avenue for new interventions. Wearable devices are increasingly used at the population level and could be an opportunity to detect individuals who will benefit most from these interventions.

In conclusion, this study identified a set of PA and SB features predicting mortality risk that might be relevant, new targets for prevention. These features relate to dimensions of movement behaviour that go beyond total duration in MVPA or SB. Whether interventions to modify these features among older adults are easier to implement and overcome existing individual^34^^,^^35^ or environmental^36^ barriers warrants further research.

## Contributors

SS conceived the idea for the study. MC, MAB, VvH, BL, and SS designed the study methodology. MC, MAB, BL, and SS did the investigation. MC, MY, VvH, MAB, PMV, and SS accessed and validated the dataset. MC, MAB, and VvH did the formal data analysis. MC, VvH, PMV, and SS curated the data. MC, BL, and SS prepared the first draft of the manuscript. All authors reviewed and edited the manuscript. MC and MAB were responsible for the figures. MC is the guarantor. SS and MAB supervised the study. SS acquired the funding for the study.

## Data sharing statement

Data, protocols, and other metadata of the Whitehall II study are available to the scientific community via the Whitehall II study data sharing portal (https://www.ucl.ac.uk/epidemiology-health-care/research/epidemiology-and-public-health/research/whitehall-ii/data-sharing). Information related to CoLaus data access is available to qualified, interested researchers at https://www.colaus-psycolaus.ch/professionals/how-to-collaborate/. All responses to data sharing requests must comply with the ethical and legal constraints of Switzerland.

## Declaration of interests

We declare no competing interests.

## Appendix A Supplementary data

Supplementary appendix_R2
