# Supplementary material for: Identification of physical activity and sedentary behaviour dimensions that predict mortality risk in older adults: Development of a machine learning model in the Whitehall II accelerometer sub-study and external validation in the CoLaus study
Source: eClinicalMedicine. 2022 Dec 13;55:101773. doi: 10.1016/j.eclinm.2022.101773 (PMC9772789; doi:10.1016/j.eclinm.2022.101773)
Supplement: Supplementary appendix_R2 [file mmc1.docx]

**SUPPLEMENTARY APPENDIX**

**Identification of physical activity and sedentary behaviour dimensions that predict mortality risk in older adults: development of a machine learning model in the Whitehall II accelerometer sub-study and external validation in the CoLaus study**

Mathilde Chen,*^1^ PhD; Benjamin Landré,*^1^ PhD; Pedro Marques-Vidal,^2^ MD, PhD; Vincent T. van Hees,^3^ PhD; April C.E. van Gennip,^4,5^ MD; Mikaela Bloomberg,^6^ PhD; Manasa S. Yerramalla,^1^ PhD; Mohamed Amine Benadjaoud,^7^ PhD; Séverine Sabia,^1,6^ PhD

^1^ Université Paris Cité, Inserm U1153, CRESS, Epidemiology of Ageing and Neurodegenerative diseases, 10 avenue de Verdun, 75010 Paris, France

^2^ Department of Medicine, Internal Medicine, Lausanne University Hospital and University of Lausanne, Switzerland

^3^ Accelting, Almere, The Netherlands

^4^ Department of Internal Medicine, Maastricht University Medical Centre, The Netherlands

^5^ School for Cardiovascular Diseases CARIM, Maastricht University, The Netherlands

^6^ Department of Epidemiology and Public Health, University College London, UK

^7^ Institute for Radiological Protection and Nuclear Safety (IRSN), Fontenay-Aux-Roses, France

*These authors contributed equally

**Corresponding author & address**

Mathilde Chen

Université Paris Cité, Inserm U1153, CRESS, Epidemiology of Ageing and Neurodegenerative diseases, 10 avenue de Verdun, 75010 Paris, France

Email: mathilde.chen@inserm.fr

[Supplementary Methods S1. Socio-demographic, behavioural, and health-related covariates in the Whitehall II accelerometer sub-study 3](#_Toc121306386)

[Supplementary Methods S2. Procedure to derive the physical activity and sedentary behaviour composite scores in Whitehall II accelerometer sub-study 4](#_Toc121306387)

[Supplementary Methods S3. Description of measures of physical activity and sedentary accelerometer-assessed variables, mortality, and covariates in the CoLaus accelerometer sub-study 5](#_Toc121306388)

[Supplementary Figure S1. Flow chart for sample selection in Whitehall II accelerometer sub-study 7](#_Toc121306397)

[Supplementary Figure S2. Correlation plot of the 21 accelerometer-derived physical activity and sedentary behaviour variables 8](#_Toc121306398)

[Supplementary Figure S3. Factor loadings of physical activity and sedentary behaviour variables, including features standardised to waking period duration, in composite scores identified as predictors of mortality risk 9](#_Toc121306399)

[Supplementary Figure S4. Flow chart for sample selection in CoLaus II accelerometer sub-study 10](#_Toc121306400)

[Supplementary Table S1. Description of the 21 accelerometer-derived daily physical activity and sedentary behaviour features 11](#_Toc121306404)

[Supplementary Table S2. Predictive performance for mortality risk of physical activity and sedentary behaviour composite scores 1 and 2 derived from features standardised to waking period duration in the Whitehall II accelerometer sub-study (N cases/N total = 410/3991, mean [standard deviation] follow-up = 8·1 [1·3] years) 13](#_Toc121306405)

[Supplementary Table S3. Baseline characteristics by subgroups in Whitehall II accelerometer sub-study 14](#_Toc121306406)

[Supplementary Table S4. Predictive performance of the physical activity and sedentary behaviour composite score 1 for mortality risk among participants aged <74 and participants aged ≥74 years in the Whitehall II accelerometer sub-study 17](#_Toc121306407)

[Supplementary Table S5. Predictive performance of the physical activity and sedentary behaviour composite score 1 for mortality risk by sex in Whitehall II accelerometer sub-study 18](#_Toc121306408)

[Supplementary Table S6. Predictive performance of physical activity and sedentary behaviour composite scores for mortality risk by body mass index status in Whitehall II accelerometer sub-study 19](#_Toc121306409)

[Supplementary Table S7. Predictive performance of physical activity and sedentary behaviour composite scores for mortality risk by morbidity status in Whitehall II accelerometer sub-study 20](#_Toc121306410)

[Supplementary Table S8. Sample characteristics in 2014-2017 by mortality status at the end of follow-up (February 2021) in CoLaus accelerometer sub-study 21](#_Toc121306411)

Supplementary Methods S1. Socio-demographic, behavioural, and health-related covariates in the Whitehall II accelerometer sub-study

Data were drawn from questionnaires and clinical evaluations conducted in 2012-2013, as well as from electronic health records (Hospital Episode Statistics (HES), cancer registry, and the Mental Health Services Data Set).

Socio-demographic factors included age, sex, ethnicity (white, non-white), marital status (married/cohabitating, divorced/widowed/single), and education level (no academic qualifications, lower secondary school, higher secondary school, university higher degree; entered as an ordinal variable).

Behavioural factors were smoking status (current, past, never smoker), alcohol consumption (none, moderate (1-14 units/week), high (>14 units/week)), and fruit and vegetable consumption (Less than daily, Daily, Twice or more daily).

Health-related factors included body mass index (categorized as <25 (normal), 25-29·9 (overweight), and ≥30 (obesity) kg/m²), hypertension (systolic/diastolic blood pressure ≥140/90 mmHg or use of antihypertensive drugs), hyperlipidemia (low-density lipoproteins >4·1 mmol/L or use of lipid-lowering drugs) assessed at the clinical examination, diabetes (fasting glucose ≥7·0 mmol/L, self-reported doctor-diagnosed diabetes, use of anti-diabetic medications, or record in HES), number of chronic diseases (including coronary heart disease, stroke, heart failure, arthritis, cancer, depression, dementia, Parkinson’s disease, and chronic obstructive pulmonary disease; assessed using HES records and data collected at Whitehall clinical exams as well as mental health records for depression and dementia), number of limited basic activities of daily living (assessed by questions on limitations in the following activities: dressing, walking, bathing, eating, getting in bed, and using the toilet), and number of limited instrumental ADL (IADL, assessed by questions on limitations in the following activities: cooking, shopping for groceries, making telephone calls, taking medication, doing housework, and managing money).^1,2^

*References*

1. Katz S, Downs TD, Cash HR, Grotz RC. Progress in Development of the Index of ADL1. The Gerontologist 1970; 10(1_Part_1): 20-30.
2. Lawton MP, Brody EM. Assessment of Older People: Self-Maintaining and Instrumental Activities of Daily Living1. The Gerontologist 1969; 9(3_Part_1): 179-86.

Supplementary Methods S2. Procedure to derive the physical activity and sedentary behaviour composite scores in Whitehall II accelerometer sub-study

We identified relevant PA and SB features for all-cause mortality in the Whitehall II accelerometer sub-study among the 21 PA and SB variables derived from accelerometer data (Supplementary Table 1). Owing to substantial correlations among these features (Supplementary Figure 2), we used a sparse Partial Least Square (SPLS) regression for censored data,^1,2^ a powerful and flexible tool to pinpoint the most important predictors among numerous interrelated variables such as accelerometer-derived features.

SPLS regression determined a decomposition of centered data matrices of the accelerometer features into several composite scores and associated loading factors. The composite scores are linear combinations of accelerometer variables and associated loading vectors, reflecting the relative importance of the variables in the model. Penalization controlled by a sparsity threshold is applied on loading vectors to perform variable selection.^2^

In order to account for the censored nature of mortality data, the SPLS regression with the null deviance residuals of an unadjusted Cox model as the outcome was used to compute the composite scores.^1^

The number of composite scores (n=2) and the sparsity threshold (λ=0·79) were determined using a 5-folds cross-validation procedure. The loads associated with selected features are presented in Figure 1 and in the following table. For replication in another cohort, these loads shall be applied to standardised values of PA and SB features based on means and standard deviations from the derivation study sample as presented in Table 2.

|  | **Associated load in** | |
| --- | --- | --- |
|  | **PA and SB**  **composite score 1** | **PA and SB**  **composite score 2** |
| **Overall activity level** |  |  |
| Mean acceleration | -0·29563527 | 0·41315872 |
| **Total duration** |  |  |
| Total duration in MVPA (min/day) | -0·20443774 | -0·45117158 |
| **Bouts duration** |  |  |
| Mean duration of SB bouts (min) | 0·30504484 | -0·08269421 |
| Time in <10 min MVPA bouts | -0·30393966 | 0·22395452 |
| Time in 10-29·9 min SB bouts | -0·30380242 | 0·23067334 |
| Time in ≥30 min SB bouts (min/day) | 0·29415912 | 0·25199440 |
| **Frequency** |  |  |
| Number of LIPA bouts (N(/day) | -0·28111674 | 0·19422558 |
| Number of MVPA bouts (N/day) | -0·30518813 | 0·23948663 |
| Number of days with ≥30 min of MVPA | -0·34108721 | -0·16977244 |
| **Intensity distribution** |  |  |
| Intensity intercept | 0·29279676 | 0·18757897 |
| Intensity gradient | -0·08233124 | -0·46960397 |
| **Timing** |  |  |
| Timing of physical activity | -0·35430572 | -0·26911872 |

Abbreviations: PA: physical activity; SB: sedentary behavior; MVPA: moderate-to-vigorous physical activity.

The analyses using the approach described above were conducted using the version 1.7.6 of the R-Package *plsRcox* (<https://cran.r-project.org/web/packages/plsRcox/index.html>).^1, 3^

*References*

1. Bastien P, Bertrand F, Meyer N, Maumy-Bertrand M. Deviance residuals-based sparse PLS and sparse kernel PLS regression for censored data. *Bioinformatics,* 2015. **31**(3), 397-404.
2. Lê Cao K-A, Martin PGP, Robert-Granié C, Besse P. Sparse canonical methods for biological data integration: application to a cross-platform study. *BMC Bioinformatics,* 2009; **10**(1): 34.
3. Bertrand F, Bastien P, Meyer N, Maumy-Bertrand M. plsRcox, Cox-Models in a high dimensional setting in R. Proceedings of User 2014!, Los Angeles, page 177.

Supplementary Methods S3. Description of measures of physical activity and sedentary accelerometer-assessed variables, mortality, and covariates in the CoLaus accelerometer sub-study

***Study population***

The CoLaus study is a population-based cohort exploring the biological, genetic, and environmental determinants of cardiovascular diseases.^1^ A nonstratified, representative sample of the population of Lausanne (Switzerland) was recruited between 2003 and 2006 based on the following inclusion criteria: (1) age 35–75 years and (2) willingness to participate. The second follow-up occurred 10 years after the baseline survey and included an accelerometer measure of physical activity and sedentary behaviour.

***Accelerometer-derived physical activity and sedentary behaviour features***

Accelerometry-based physical activity and sedentary behaviour were assessed using a wrist-worn triaxial accelerometer (GENEActiv, Activinsights Ltd, UK). The accelerometers were pre-programmed with a 50 Hz sampling frequency and subsequently attached to the participants’ right wrist. Participants were requested to wear the device continuously for 14 days over 24 hours in their free-living conditions. Data were extracted from accelerometers using the GENEActiv software version 2.9 (GENEActiv, Activinsights Ltd., United Kingdom) and processed using GGIR R package (version 2.3-3).^2^ Accelerometer data were collapsed into 60-second epoch files. Sleep periods were detected using a validated algorithm,^2^ without sleeplog. Waking periods from day 2 through day 13 were used, resulting in 11 full days of data. Variables similar to those in the Whitehall II accelerometer sub-study were derived as described in eTable 1.

***Mortality***

All deaths up to February 2021 were adjudicated by an independent panel of internal medicine physicians, blinded to all study variables.

***Socio-demographic, behavioural, and health-related covariates***

Data on socio-demographic, behavioural, and health-related factors were drawn from questionnaires and clinical examination.^1,3,4^

Socio-demographic factors included age, sex, ethnicity, education (completed mandatory education, apprenticeship, high school, or university), and marital status (married/cohabitating, or single/divorced/windowed).

Behavioural factors were smoking status (current, past, never smoker), alcohol intake (none, moderate (1-14 units/week), high (>14 units/week)), and fruit and vegetable intake (Less than daily, Daily, Twice or more daily).

Health-related factors included body mass index (categorized as <25 (normal), 25-29·9 (overweight), and ≥30 (obesity), in kg/m²), hypertension (systolic/diastolic blood pressure ≥140/90 mmHg or use of antihypertensive drugs), hyperlipidemia (low-density lipoproteins >4·1 mmol/L or use of lipid-lowering drugs) assessed at the clinical examination, diabetes (fasting glucose ≥7·0 mmol/L or use of anti-diabetic medications), number of chronic diseases (including coronary heart disease, stroke, heart failure [self-reported by the participants and verified and medically documented by a trained investigator, and further validated using pre-defined criteria by an independent adjudication committee composed of internists, cardiologists and a neurologist], and depression [assessed by the Center for Epidemiological Studies-Depression Scale ≥17 for men and ≥23 for women])^5^, presence of limited basic activities of daily living (assessed by the question: “The last 4 weeks, did you feel difficult to take care of you?”), and presence of limited basic instrumental activities of daily living (assessed by the question: “The last 4 weeks, did you feel difficult to go shopping?”).

*References*

1. Firmann, M. et al. The CoLaus study: a population-based study to investigate the epidemiology and genetic determinants of cardiovascular risk factors and metabolic syndrome. BMC Cardiovascular Disorders 8, 6, doi:10.1186/1471-2261-8-6 (2008).
2. Migueles, J. H., Rowlands, A. V., Huber, F., Sabia, S. & van Hees, V. T. GGIR: A research community–driven open Source R package for generating physical activity and sleep outcomes from multi-day raw accelerometer data. Journal for the Measurement of Physical Behaviour 2, 188-196, doi:10.1123/jmpb.2018-0063 (2019).
3. Morabia A, Bernstein M, Kumanyika S, et al. [Development and validation of a semi-quantitative food questionnaire based on a population survey]. Sozial- und Präventivmedizin 1994; 39(6): 345-69
4. Bernstein M, Huot I, Morabia A. Amélioration des performances d'un questionnaire alimentaire semi-quantitatif comparé à un rappel des 24 heures. Sante Publique 1995; 4: 403-13.
5. Fuhrer R, Rouillon F. La version française de l'échelle CES-D (Center for Epidemiologic Studies-Depression Scale). Description et traduction de l'échelle d'autoévaluation. Psychiatrie et Psychobiologie 1989; 4: 163-6.

Supplementary Figure S1. Flow chart for sample selection in Whitehall II accelerometer sub-study

Study wave 2012-2013

N = 6308

Invited to accelerometer sub-study

N = 4880

Consented

N = 4492

No contraindications

N = 4282

Returned accelerometers

N = 4267

Valid accelerometer data*

N = 4008

**Analytical sample**

**N = 3991**

**N deaths = 410**

**Mean (standard deviation) follow-up:**

**8·3 (1·3) years**

Not invited to accelerometer sub-study due to their place of residence

N = 1428

Did not consent

N = 388

Contraindications:

Allergy to plastic or metal (n = 40)

Travelling abroad (n = 168)

Other reasons (strap too short,

cognitive impairment) (n = 2)

N = 210

Had contraindications, N = 210

Allergy to plastic or metal (n = 40)

Travelling abroad (n = 168)

Other reasons (strap too short,

cognitive impairment) (n = 2)

Accelerometers lost in the post

N = 15

Invalid accelerometer data:

Devices had no data at all (n = 68)

Devices stopped recording (n = 93)

Devices had calibration issues (n = 14)

Significant non-wear time (n = 86)

N = 261

Missing covariates at study wave 2012-2013

N = 17

Legend: *Valid data is defined as daily wear time ≥2/3 of waking hours, for at least 2 weekdays and 2 week-end days.

Supplementary Figure S2. Correlation plot of the 21 accelerometer-derived physical activity and sedentary behaviour variables


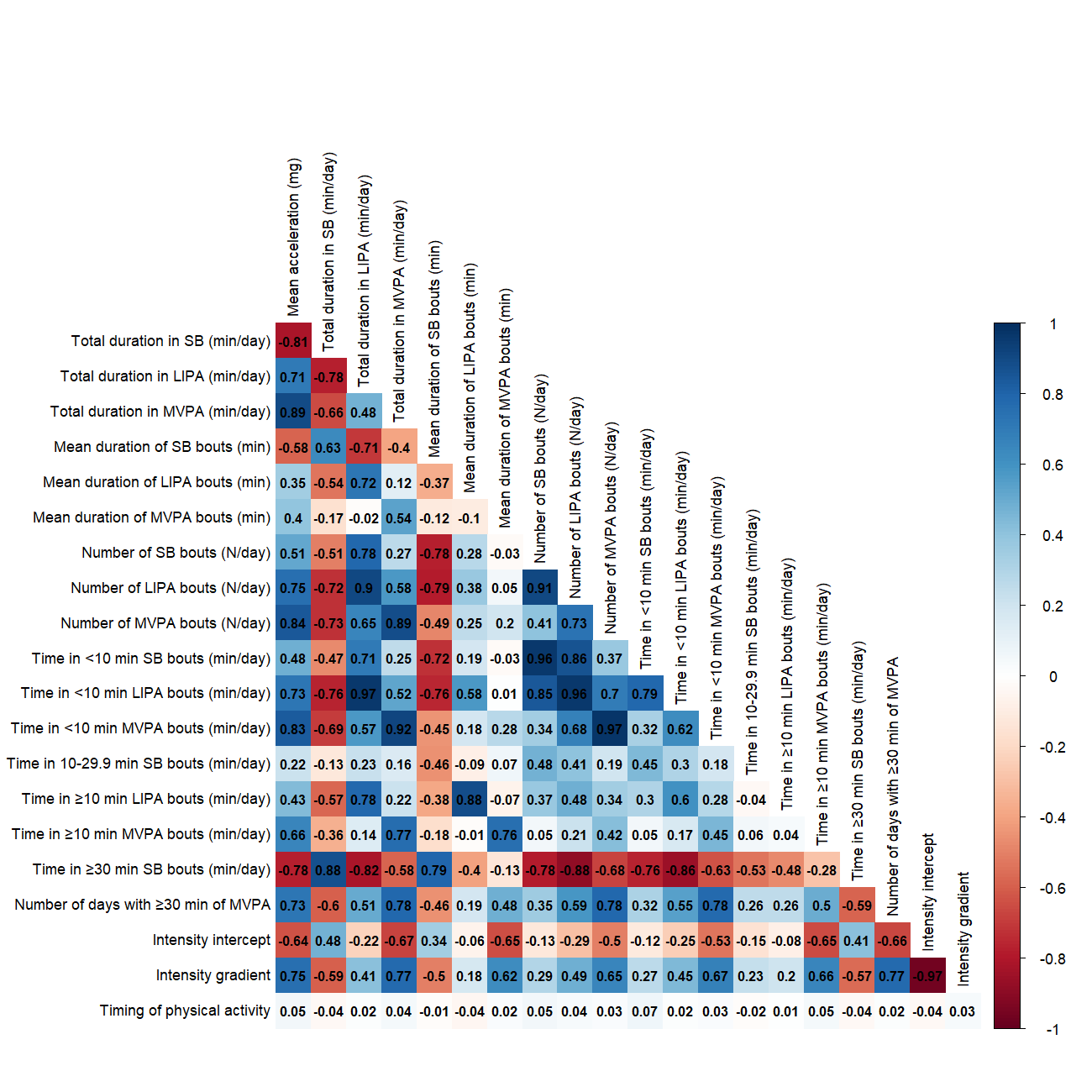


Legend: MVPA: moderate-to-vigorous physical activity; PA: physical activity; LIPA: light-intensity physical activity; SB: sedentary behaviour. Colour of each cell reflects the direction and strength of correlation between two features

Supplementary Figure S3. Factor loadings of physical activity and sedentary behaviour variables, including features standardised to waking period duration, in composite scores identified as predictors of mortality risk


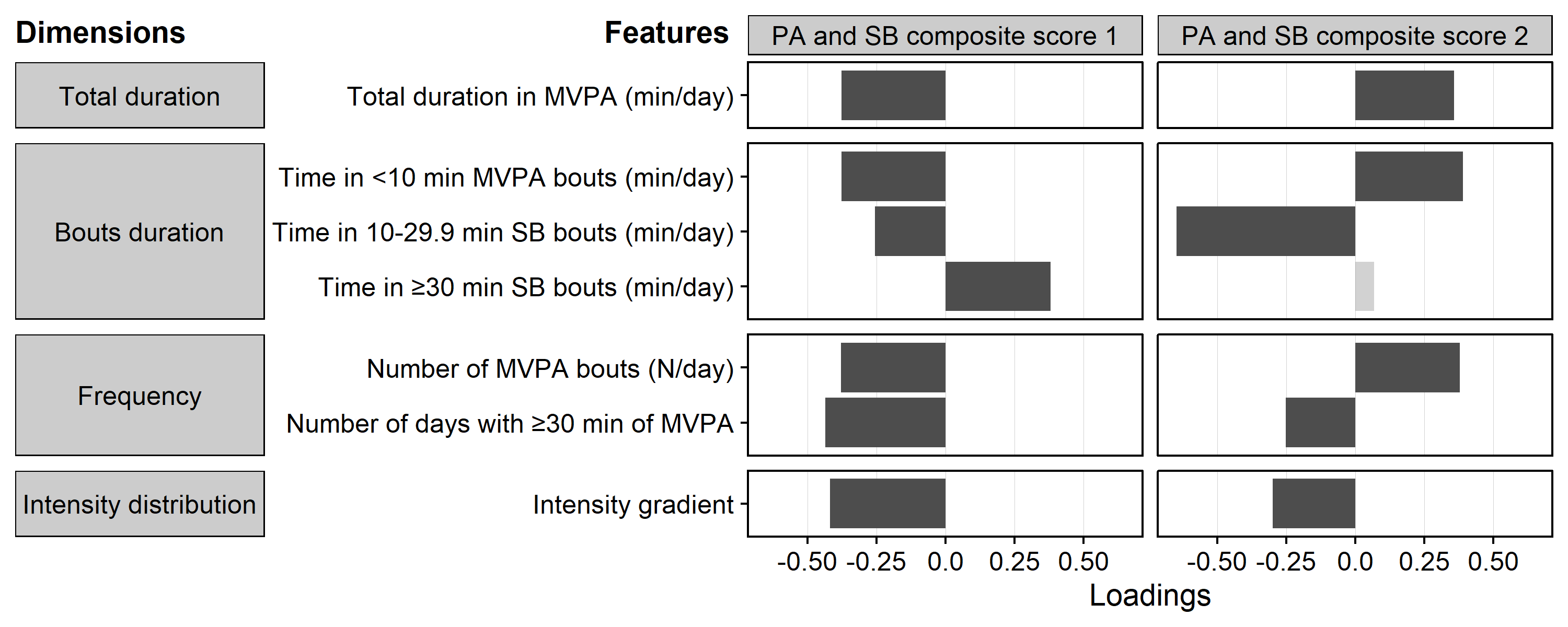


Legend: PA: physical activity; SB: sedentary behaviour; MVPA: moderate-to-vigorous physical activity; LIPA: light-intensity physical activity.

Dark grey: factor loading absolute value ≥0·2; light grey: factor loading absolute value <0·2. Features that were standardised to waking period duration are presented in Supplementary Table 1

Supplementary Figure S4. Flow chart for sample selection in CoLaus II accelerometer sub-study

Study wave 2014-2017

N = 4881

Participants aged ≥ 60 years

N = 2802

Returned accelerometers

N = 1648

Valid accelerometer data*

N = 1599

Participants with full set of covariates

N = 1582

Participants <60 years old

N = 2079

Did not take part to the accelerometer sub-study

N = 1154

Invalid accelerometer data

N = 49

Missing covariates at study wave 2014-2017

N = 17

**Analytical sample**

**N = 1329, N deaths = 105**

**Mean (standard deviation) follow-up:**

**3·8 (0·7) years**

Missing vital status

N = 253

Legend: *Valid data is defined as daily wear time ≥2/3 of waking hours, for at least 2 weekdays and 2 week-end days.

Supplementary Table S1. Description of the 21 accelerometer-derived daily physical activity and sedentary behaviour features

| **Dimension** | **Feature** | **Name in GGIR (version 2.3-3)** | **Description** |
| --- | --- | --- | --- |
| Overall activity  level | Mean acceleration | ACC_day_mg_wei | Overall activity level during waking hours, defined as average acceleration during waking period (m*g*) |
| Total  duration | Total duration in SB * | dur_day_total_IN_min_wei | Total daily time during waking period (min/day) spent in 60s-epoch acceleration<40 m*g* for SB, 40-99 m*g* for LIPA, ≥100 m*g* for MVPA |
|  | Total duration in LIPA * | dur_day_total_LIG_min_wei |  |
|  | Total duration in MVPA * | Derived as: dur_day_total_MOD_min_wei + dur_day_total_VIG_min_wei |  |
| Bouts  duration | Mean duration of SB bouts | FRAG_mean_dur_IN_day_wei | Average duration of bouts in SB, LIPA, and MVPA,  computed as the total daily duration during waking period divided by daily number of bouts |
|  | Mean duration of LIPA bouts | FRAG_mean_dur_LIPA_day_wei |  |
|  | Mean duration of MVPA bouts | FRAG_mean_dur_MVPA_day_wei |  |
|  | Time in <10 min SB bouts * | dur_day_IN_unbt_min_wei | Daily time spent in SB, LIPA, and MVPA bouts of different length during waking period |
|  | Time in 10-30 min SB bouts * | dur_day_IN_bts_10_30_min_wei |  |
|  | Time in ≥30 min SB bouts * | dur_day_IN_bts_30_min_wei |  |
|  | Time in <10 min LIPA bouts * | dur_day_LIG_unbt_min_wei |  |
|  | Time in ≥10 min LIPA bouts * | dur_day_LIG_bts_10_min_wei |  |
|  | Time in <10 min MVPA bouts * | Derived as: (dur_day_total_MOD_min_wei + dur_day_total_VIG_min_wei) - dur_day_MVPA_bts_10_min_wei |  |
|  | Time in ≥10 min MVPA bouts * | dur_day_MVPA_bts_10_min_wei |  |
| Frequency | Number of SB bouts | FRAG_Nfrag_IN_day_wei | Total daily number of bouts in SB, LIPA, and MVPA during waking period |
|  | Number of LIPA bouts | FRAG_Nfrag_LIPA_day_wei |  |
|  | Number of MVPA bouts | Derived as: Nblocks_day_total_MOD_wei + Nblocks_day_total_VIG_wei |  |
|  | Number of days with ≥30 min of MVPA | Derived from day-level summary using: dur_day_total_MOD_min + dur_day_total_VIG_min | The number of days with total duration in MVPA ≥30 min, representing the number of days meeting physical activity guidelines^1^ |
| Intensity distribution^2^ | Intensity intercept | ig_gradient_wei | Intercept and slope of the linear regression between log of daily intensity and log of time in that intensity across waking period, averaged over the days of the observational period  Examples: Higher intercept and steeper gradient (i.e. more negative gradient) represent more time in sedentary SB and little time spent in midrange and higher intensity. Lower intercept and shallow gradient correspond to more time spread across the range of intensities. |
|  | Intensity gradient | ig_intercept_wei |  |
| Timing | Timing of PA | M5TIME_num_wei | Start of the most active five consecutive hours, averaged over the observational period. Example: 7 = the most active five consecutive hours starts at 7:00am until noon |

Abbreviations: SB: sedentary behaviour; LIPA: light-intensity physical activity; MVPA: moderate-to-vigorous physical activity.

*Features that were standardised on mean waking period duration in sensitivity analyses (standardised value=original value x 960 / waking period duration, 16 hours (960 minutes) being the mean waking period duration in the study sample).

Notes: Average 60-s epoch acceleration <40 milligravity (m*g*) during waking period was classified as SB, 40-99 m*g* as light-intensity PA (LIPA), and ≥100 m*g* as MVPA.

As usually done in previous studies, we examined MVPA, not separating moderate from vigorous physical activity. For information, only 79 participants reached 10 min of daily vigorous physical activity in the Whitehall sample.

A bout corresponds to an uninterrupted episode spent in a given range of acceleration (<40 m*g* for SB, 40-99 m*g* for LIPA, ≥100 m*g* for MVPA).

All features were computed at an individual level over daily waking periods and averaged over seven days. For those with valid data (i.e. defined as daily wear time ≥2/3 of waking hours, for at least 2 weekdays and 2 week-end days) for less than seven days, a weighted average was computed using information on the number of week and weekend days,^3^ with the exception of the number of days meeting physical activity guidelines which was normalised to account for the total number of days with valid data.

*References*

1. American College of Sports Medicine. Physical Activity Guidelines. 2018. <https://www.acsm.org/education-resources/trending-topics-resources/physical-activity-guidelines>.

2. Rowlands AV, Edwardson CL, Davies MJ, Khunti K, Harrington DM, Yates T. Beyond Cut Points: Accelerometer Metrics that Capture the Physical Activity Profile. *Med Sci Sports Exerc* 2018; **50**(6): 1323-32.

3. Sabia S, van Hees VT, Shipley MJ, Trenell MI, Hagger-Johnson G, Elbaz A, Kivimaki M, Singh-Manoux A. Association Between Questionnaire- and Accelerometer-Assessed Physical Activity: The Role of Sociodemographic Factors, *American Journal of Epidemiology*, 2014; **179**(6): 781–90

Supplementary Table S2. Predictive performance for mortality risk of physical activity and sedentary behaviour composite scores 1 and 2 derived from features standardised to waking period duration in the Whitehall II accelerometer sub-study (N cases/N total = 410/3991, mean [standard deviation] follow-up = 8·1 [1·3] years)

|  | **HR (95% CI)**  **PA and SB composite scores** | **Royston’s R²**  **(95% CI)** | **AIC**  **(95% CI)** | **ΔAIC**  **(95% CI)** | **ΔAIC**  **(95% CI)** | **Sensitivity %**  **(95% CI) ^d^** | **Specificity %**  **(95% CI) ^d^** | **C-index**  **(95% CI)** | **ΔC-index**  **(95% CI)** | **ΔC-index**  **(95% CI)** |
| --- | --- | --- | --- | --- | --- | --- | --- | --- | --- | --- |
| **Model 1 ^a^** | NA | 0·366  (0·328, 0·424) | 6374·6  (5801·3, 6989·1) | *Ref·* | NA | 69·9  (56·4, 87·9) | 66·4  (47·1, 82·3) | 0·751  (0·731, 0·778) | *Ref·* | NA |
| **Model 2 ^b^** | PA and SB score 1:  1·14 (1·08, 1·20), p<0·001 | 0·384  (0·344, 0·441) | 6352·5  (5790·7, 6959·0) | -22·0  (-49·4, -7·6) * | *Ref·* | 69·4  (51·5, 84·3) | 70·4  (52·5, 84·7) | 0·758  (0·736, 0·784) | 0·007  (0·002, 0·015) * | *Ref·* |
| **Model 3 ^c^** | PA and SB score 1:  1·14 (1·08, 1·21), p<0·001 | 0·384  (0·344, 0·442) | 6354·5  (5791·6, 6960·6) | -20·0  (-48·8, -6·8) * | 2·0  (-8·4, 2·0) | 69·4  (51·7, 84·4) | 70·4  (53·0, 85·5) | 0·758  (0·737, 0·784) | 0·007  (0·002, 0·015) * | -0·00001  (-0·0003, 0·004) |
|  | PA and SB score 2:  1·00 (0·89, 1·11), p=0·95 |  |  |  |  |  |  |  |  |  |

Abbreviations: CI: confidence interval; AIC: Akaike information criterion; C-index: Harrell’s C-index

* Confidence intervals (calculated based on 1000 bootstrap samples) not containing zero indicate significant differences in predictive performance between Model 1 and Model 2, and between Model 2 and Model 3

^a^ Model 1: Model includes socio-demographic, behavioural, and health-related factors

^b^ Model 2: Model 1 additionally adjusted for the physical activity and sedentary behaviour composite score 1

^c^ Model 3: Model 2 additionally adjusted for the physical activity and sedentary behaviour composite score 2

^d^ Youden index cutoff points for the calculation of the sensitivity and specificity: 0·363 (95% CI: [0·301, 0·475]) for model 1, 0·374 (95% CI: [0·318, 0·489]) for model 2, and 0·381 (95% CI: [0·354, 0·424]) for Model 3

Supplementary Table S3. Baseline characteristics by subgroups in Whitehall II accelerometer sub-study

|  | **Age groups** | |  | **Sex groups** | |  | **Body mass index groups** | | |  | **Morbidity groups** | |
| --- | --- | --- | --- | --- | --- | --- | --- | --- | --- | --- | --- | --- |
|  | **<74 years (N=3001)** | **≥74 years (N=990)** |  | **Men (N=2961)** | **Women (N=1030)** |  | **Normal (N=1548)** | **Overweight (N=1720)** | **Obesity (N=723)** |  | **None (N=2362)** | **One or more (N=1629)** |
| **Socio-demographic factors** |  |  |  |  |  |  |  |  |  |  |  |  |
| Age (years), M (SD) | 66·7 (3·6) | 77·5 (2·2) |  | 69·4 (5·7) | 69·5 (5·8) |  | 69·5 (5·8) | 69·5 (5·6) | 68·9 (5·6) |  | 68·5 (5·5) | 70·7 (5·8) |
| Women | 770 (25·7) | 260 (26·3) |  | / | / |  | 419 (27·1) | 349 (20·3) | 262 (36·2) |  | 545 (23·1) | 485 (29·8) |
| Non-white ethnicity | 205 (6·8) | 90 (9·1) |  | 164 (5·5) | 131 (12·7) |  | 110 (7·1) | 119 (6·9) | 66 (9·1) |  | 155 (6·6) | 140 (8·6) |
| Education |  |  |  |  |  |  |  |  |  |  |  |  |
| No academic qualifications | 208 (6·9) | 162 (16·4) |  | 191 (6·5) | 179 (17·4) |  | 115 (7·4) | 170 (9·9) | 85 (11·8) |  | 173 (7·3) | 197 (12·1) |
| Lower secondary school | 908 (30·3) | 364 (36·8) |  | 912 (30·8) | 360 (35·0) |  | 474 (30·6) | 574 (33·4) | 224 (31·0) |  | 736 (31·2) | 536 (32·9) |
| Higher secondary school | 882 (29·4) | 229 (23·1) |  | 857 (28·9) | 254 (24·7) |  | 395 (25·5) | 484 (28·1) | 232 (32·1) |  | 661 (28·0) | 450 (27·6) |
| University | 735 (24·5) | 180 (18·2) |  | 732 (24·7) | 183 (17·8) |  | 407 (26·3) | 357 (20·8) | 151 (20·9) |  | 577 (24·4) | 338 (20·7) |
| Higher degree | 268 (8·9) | 55 (5·6) |  | 269 (9·1) | 54 (5·2) |  | 157 (10·1) | 135 (7·8) | 31 (4·3) |  | 215 (9·1) | 108 (6·6) |
| Not married/cohabitating | 706 (23·5) | 304 (30·7) |  | 519 (17·5) | 491 (47·7) |  | 391 (25·3) | 385 (22·4) | 234 (32·4) |  | 550 (23·3) | 460 (28·2) |
| **Behavioural factors** |  |  |  |  |  |  |  |  |  |  |  |  |
| Smoking status |  |  |  |  |  |  |  |  |  |  |  |  |
| Never smoker | 1487 (49·6) | 474 (47·9) |  | 1370 (46·3) | 591 (57·4) |  | 816 (52·7) | 807 (46·9) | 338 (46·7) |  | 1229 (52·0) | 732 (44·9) |
| Past smoker | 1325 (44·2) | 484 (48·9) |  | 1421 (48·0) | 388 (37·7) |  | 659 (42·6) | 806 (46·9) | 344 (47·6) |  | 1009 (42·7) | 800 (49·1) |
| Current smoker | 189 (6·3) | 32 (3·2) |  | 170 (5·7) | 51 (5·0) |  | 73 (4·7) | 107 (6·2) | 41 (5·7) |  | 124 (5·2) | 97 (6·0) |
| Alcohol intake |  |  |  |  |  |  |  |  |  |  |  |  |
| 0 unit/week | 581 (19·4) | 231 (23·3) |  | 466 (15·7) | 346 (33·6) |  | 304 (19·6) | 313 (18·2) | 195 (27·0) |  | 448 (19·0) | 364 (22·3) |
| 1-14 units/week | 1667 (55·5) | 587 (59·3) |  | 1668 (56·3) | 586 (56·9) |  | 919 (59·4) | 983 (57·2) | 352 (48·7) |  | 1335 (56·5) | 919 (56·4) |
| > 14 units/week | 753 (25·1) | 172 (17·4) |  | 827 (27·9) | 98 (9·5) |  | 325 (21·0) | 424 (24·7) | 176 (24·3) |  | 579 (24·5) | 346 (21·2) |
| Fruit and vegetable intake |  |  |  |  |  |  |  |  |  |  |  |  |
| Less than daily | 622 (20·7) | 204 (20·6) |  | 644 (21·7) | 182 (17·7) |  | 277 (17·9) | 375 (21·8) | 174 (24·1) |  | 474 (20·1) | 352 (21·6) |
| Daily | 597 (19·9) | 255 (25·8) |  | 682 (23·0) | 170 (16·5) |  | 298 (19·3) | 390 (22·7) | 164 (22·7) |  | 488 (20·7) | 364 (22·3) |
| Twice daily or more | 1782 (59·4) | 531 (53·6) |  | 1635 (55·2) | 678 (65·8) |  | 973 (62·9) | 955 (55·5) | 385 (53·3) |  | 1400 (59·3) | 913 (56·0) |
|  |  |  |  |  |  |  |  |  |  |  |  |  |
|  |  |  |  |  |  |  |  |  |  |  |  |  |
| **Health-related factors** |  |  |  |  |  |  |  |  |  |  |  |  |
| Body mass index |  |  |  |  |  |  |  |  |  |  |  |  |
| Normal (< 25 kg/m²) | 1147 (38·2) | 401 (40·5) |  | 1129 (38·1) | 419 (40·7) |  | / | / | / |  | 1011 (42·8) | 537 (33·0) |
| Overweight (25-29·9 kg/m²) | 1289 (43·0) | 431 (43·5) |  | 1371 (46·3) | 349 (33·9) |  | / | / | / |  | 975 (41·3) | 745 (45·7) |
| Obesity (≥ 30 kg/m²) | 565 (18·8) | 158 (16·0) |  | 461 (15·6) | 262 (25·4) |  | / | / | / |  | 376 (15·9) | 347 (21·3) |
| Hypertension | 1403 (46·8) | 663 (67·0) |  | 1586 (53·6) | 480 (46·6) |  | 611 (39·5) | 959 (55·8) | 496 (68·6) |  | 1082 (45·8) | 984 (60·4) |
| Hyperlipidemia | 1456 (48·5) | 565 (57·1) |  | 1500 (50·7) | 521 (50·6) |  | 654 (42·2) | 935 (54·4) | 432 (59·8) |  | 1056 (44·7) | 965 (59·2) |
| Prevalent diabetes | 355 (11·8) | 159 (16·1) |  | 381 (12·9) | 133 (12·9) |  | 125 (8·1) | 229 (13·3) | 160 (22·1) |  | 248 (10·5) | 266 (16·3) |
| Number of chronic diseases,^a^ M (SD) | 0·5 (0·7) | 0·8 (0·9) |  | 0·5 (0·7) | 0·6 (0·8) |  | 0·5 (0·7) | 0·6 (0·8) | 0·6 (0·8) |  | 0·0 (0·0) | 1·3 (0·6) |
| Number of basic ADL,^b^ M (SD) | 0·1 (0·6) | 0·3 (0·9) |  | 0·2 (0·6) | 0·2 (0·7) |  | 0·1 (0·6) | 0·2 (0·6) | 0·3 (0·9) |  | 0·1 (0·4) | 0·3 (0·9) |
| Number of IADL,^c^ M (SD) | 0·2 (0·6) | 0·3 (0·8) |  | 0·2 (0·5) | 0·3 (0·8) |  | 0·2 (0·6) | 0·2 (0·6) | 0·3 (0·7) |  | 0·1 (0·5) | 0·3 (0·8) |
| **Accelerometer variables** |  |  |  |  |  |  |  |  |  |  |  |  |
| ***Overall activity level****, M (SD)* |  |  |  |  |  |  |  |  |  |  |  |  |
| Mean acceleration (m*g*) | 33·2 (9·8) | 27·5 (7·9) |  | 31·9 (9·7) | 31·6 (9·7) |  | 34·2 (10·7) | 31·4 (8·7) | 27·8 (7·9) |  | 33·0 (9·9) | 30·1 (9·1) |
| ***Total duration (min)****, M (SD)* |  |  |  |  |  |  |  |  |  |  |  |  |
| Sedentary behaviour | 707·1 (97·8) | 750·7 (100·2) |  | 721·5 (98·3) | 707·5 (104·6) |  | 697·3 (101·4) | 721·3 (96·0) | 754·1 (95·8) |  | 710·4 (98·5) | 728·8 (101·5) |
| Light intensity physical activity | 215·0 (66·1) | 196·1 (75·7) |  | 205·4 (66·4) | 224·4 (74·6) |  | 220·0 (70·8) | 210·1 (67·2) | 190·1 (65·3) |  | 214·6 (67·6) | 204·1 (70·7) |
| Moderate-to-vigorous physical activity | 62·6 (39·0) | 36·3 (29·5) |  | 58·3 (38·4) | 49·8 (38·2) |  | 64·8 (41·6) | 54·7 (36·6) | 40·8 (30·3) |  | 61·3 (38·7) | 48·5 (37·0) |
| ***Bouts duration****, M (SD)* |  |  |  |  |  |  |  |  |  |  |  |  |
| Mean duration (min) of activity bout of |  |  |  |  |  |  |  |  |  |  |  |  |
| Sedentary behaviour | 10·9 (5·1) | 13·1 (7·6) |  | 11·6 (6·1) | 11·0 (5·2) |  | 10·8 (6·3) | 11·4 (5·1) | 13·0 (6·6) |  | 11·0 (4·8) | 12·2 (7·2) |
| Light intensity physical activity | 2·4 (0·3) | 2·4 (0·4) |  | 2·4 (0·3) | 2·5 (0·4) |  | 2·4 (0·4) | 2·4 (0·4) | 2·4 (0·4) |  | 2·4 (0·4) | 2·4 (0·4) |
| Moderate-to-vigorous physical activity | 2·4 (0·9) | 2·0 (0·8) |  | 2·4 (0·9) | 2·1 (0·8) |  | 2·5 (1·0) | 2·3 (0·8) | 2·0 (0·8) |  | 2·4 (0·9) | 2·1 (0·9) |
| Time (min/day) in < 10 min bouts of |  |  |  |  |  |  |  |  |  |  |  |  |
| Sedentary behaviour | 149·9 (39·8) | 138·7 (43·1) |  | 145·5 (40·4) | 152·0 (42·1) |  | 152·6 (40·1) | 146·4 (40·1) | 137·2 (42·9) |  | 149·8 (40·0) | 143·3 (42·1) |
| Light intensity physical activity | 188·6 (51·9) | 169·9 (58·1) |  | 181·2 (53·0) | 192·0 (56·6) |  | 192·3 (55·0) | 183·3 (52·3) | 167·6 (52·8) |  | 188·0 (52·5) | 178·1 (55·9) |
| Moderate-to-vigorous physical activity | 47·8 (27·7) | 30·2 (22·8) |  | 44·5 (27·4) | 40·3 (28·2) |  | 48·4 (28·5) | 43·1 (27·2) | 33·6 (23·7) |  | 46·7 (27·4) | 38·7 (27·4) |
|  |  |  |  |  |  |  |  |  |  |  |  |  |
| Time (min/day) in ≥ 10 min bouts of |  |  |  |  |  |  |  |  |  |  |  |  |
| Light intensity physical activity | 26·4 (20·2) | 26·2 (24·4) |  | 24·3 (19·5) | 32·4 (24·8) |  | 27·7 (22·2) | 26·8 (21·5) | 22·5 (18·0) |  | 26·6 (21·4) | 26·0 (21·1) |
| Moderate-to-vigorous physical activity | 14·8 (18·2) | 6·1 (11·1) |  | 13·7 (17·3) | 9·5 (16·3) |  | 16·3 (20·5) | 11·6 (15·2) | 7·2 (11·1) |  | 14·6 (18·3) | 9·8 (14·8) |
| Time (min/day) in 10-29·9 min bouts of |  |  |  |  |  |  |  |  |  |  |  |  |
| Sedentary behaviour | 190·1 (43·7) | 172·4 (47·5) |  | 186·8 (45·9) | 182·8 (43·4) |  | 186·6 (43·7) | 186·9 (45·0) | 181·4 (48·9) |  | 189·2 (44·3) | 180·8 (46·3) |
| Time (min/day) in ≥ 30 min bouts of |  |  |  |  |  |  |  |  |  |  |  |  |
| Sedentary behaviour | 367·0 (135·7) | 439·6 (150·7) |  | 389·3 (141·6) | 372·7 (146·6) |  | 358·1 (139·7) | 388·0 (137·3) | 435·5 (149·1) |  | 371·4 (136·8) | 404·7 (149·5) |
| ***Frequency****, M (SD)* |  |  |  |  |  |  |  |  |  |  |  |  |
| Number (N/day) of bouts of |  |  |  |  |  |  |  |  |  |  |  |  |
| Sedentary behaviour | 73·0 (15·3) | 68·3 (17·6) |  | 71·3 (15·8) | 73·5 (16·3) |  | 73·9 (15·7) | 71·7 (15·7) | 67·7 (16·7) |  | 72·9 (15·3) | 70·2 (16·8) |
| Light intensity physical activity | 88·4 (20·7) | 78·8 (22·8) |  | 85·4 (21·4) | 87·6 (22·2) |  | 89·6 (21·5) | 85·8 (20·9) | 78·8 (21·8) |  | 88·0 (20·7) | 83·0 (22·5) |
| Moderate-to-vigorous physical activity | 25·4 (13·4) | 16·8 (11·8) |  | 23·6 (13·4) | 22·1 (14·0) |  | 25·6 (14·0) | 23·1 (13·3) | 18·6 (12·0) |  | 24·9 (13·4) | 20·9 (13·4) |
| Number of days with ≥30 min of  moderate-to-vigorous physical activity | 5·0 (2·3) | 3·1 (2·6) |  | 4·7 (2·4) | 4·2 (2·7) |  | 5·0 (2·3) | 4·5 (2·5) | 3·5 (2·6) |  | 4·9 (2·3) | 4·0 (2·7) |
| ***Intensity distribution****, M (SD)* |  |  |  |  |  |  |  |  |  |  |  |  |
| Intensity intercept | 12·3 (0·6) | 12·7 (0·6) |  | 12·3 (0·7) | 12·5 (0·6) |  | 12·2 (0·7) | 12·4 (0·6) | 12·6 (0·6) |  | 12·3 (0·7) | 12·5 (0·7) |
| Intensity gradient | -2·05 (0·20) | -2·19 (0·22) |  | -2·07 (0·22) | -2·11 (0·22) |  | -2·04 (0·22) | -2·09 (0·20) | -2·17 (0·22) |  | -2·05 (0·21) | -2·13 (0·22) |
| ***Timing****, ^d^ M (SD)* |  |  |  |  |  |  |  |  |  |  |  |  |
| Timing of physical activity (hours) | 10h 18min  (1h 36min) | 9h 54min  (1h 36min) |  | 10h 12min  (1h 36min) | 10h 12min (1h 36min) |  | 10h 18min  (1h 36min) | 10h 12min  (1h 36min) | 10h 6min  (1h 36min) |  | 10h 18min  (1h 36min) | 10h 12min  (1h 36min) |

Data are N (%), otherwise specified. Abbreviations: M: mean; SD: standard deviation; ADL: activities of daily living; IADL: instrumental activities of daily living.

^a^ Chronic disease include coronary heart disease, stroke, heart failure, arthritis, cancer, depression, dementia, Parkinson’s disease, and chronic obstructive pulmonary disease.

^b^ Basic ADL include difficulty in dressing, walking, bathing, eating, getting in bed, and using the toilet.

^c^ IADL include difficulty in cooking, shopping for groceries, making telephone calls, taking medication, doing housework, and managing money.

^d^ Timing of the five most active hours during the waking period.

Supplementary Table S4. Predictive performance of the physical activity and sedentary behaviour composite score 1 for mortality risk among participants aged <74 and participants aged ≥74 years in the Whitehall II accelerometer sub-study

|  | **HR (95% CI) of**  **the PA and SB**  **composite score 1** | **Royston’s R²**  **(95% CI)** | **AIC**  **(95% CI)** | **ΔAIC**  **(95% CI)** | **Sensitivity %**  **(95% CI) ^c^** | **Specificity %**  **(95% CI) ^c^** | **C-index**  **(95% CI)** | **ΔC-index**  **(95% CI)** |
| --- | --- | --- | --- | --- | --- | --- | --- | --- |
| **Participants aged <74 years (N cases/ N total = 187/3001)** | | | | | | | | |
| Model 1 ^a^ | *NA* | 0·304  (0·254, 0·406) | 2866·4  (2461·8, 3223·4) | *Ref.* | 55·6  (46·6, 84·0) | 82·9  (51·8, 91·5) | 0·712  (0·687, 0·760) | *Ref.* |
| Model 2 ^b^ | 1·12 (1·06, 1·18), p<0·001 | 0·331  (0·280, 0·427) | 2852·7  (2451·4, 3205·5) | -13·7  (-37·1, -3·0) * | 52·4  (44·8, 80·2) | 88·6  (57·9, 94·4) | 0·724  (0·701, 0·771) | 0·012  (0·003, 0·031) * |
| **Participants aged ≥74 years (N cases/ N total = 223/990)** | | | | | | | | |
| Model 1 ^a^ | *NA* | 0·177  (0·148, 0·288) | 2958·3  (2632·0, 3343·9) | *Ref.* | 52·6  (37·9, 85·5) | 85·1  (46·4, 95·5) | 0·655  (0·636, 0·705) | *Ref.* |
| Model 2 ^b^ | 1·08 (1·02, 1·14), p=0·005 | 0·192  (0·165, 0·313) | 2952·5  (2624·0, 3332·6) | -5·8  (-23·9, 0·8) | 63·4  (41·7, 85·8) | 76·6  (50·3, 95·7) | 0·660  (0·644, 0·710) | 0·005  (-0·001, 0·021) |

Abbreviations: HR: Hazard ratio; CI: confidence interval; AIC: Akaike information criterion; C-index: Harrell’s C-index; PA, physical activity; SB, sedentary behaviour.

* Confidence interval not containing zero indicates significant difference in predictive performance between Model 1 and Model 2

^a^ Model 1: Model includes socio-demographic, behavioural, and health-related factors

^b^ Model 2: Model 1 additionally adjusted for the physical activity and sedentary behaviour composite score 1

^c^ Youden index cutoff points for the calculation of the sensitivity and specificity are as follows: among those aged <74 years: 0·323 (95% CI: [0·285, 0·396]) for Model 1 and 0·410 (95% CI: [0·319, 0·521]) for Model 2; among those aged ≥74 years: 0·377 (95% CI: [0·253, 0·527]) for Model 1 and 0·400 (95% CI: [0·298, 0·555]) for Model 2

Supplementary Table S5. Predictive performance of the physical activity and sedentary behaviour composite score 1 for mortality risk by sex in Whitehall II accelerometer sub-study

|  | **HR (95% CI) of**  **the PA and SB**  **composite score 1** | **Royston’s R²**  **(95% CI)** | **AIC**  **(95% CI)** | **ΔAIC**  **(95% CI)** | **Sensitivity %**  **(95% CI) ^c^** | **Specificity %**  **(95% CI) ^c^** | **C-index**  **(95% CI)** | **ΔC-index**  **(95% CI)** |
| --- | --- | --- | --- | --- | --- | --- | --- | --- |
| **Men (N cases/N total = 318/2961)** | | | | | | | | |
| Model 1 ^a^ | *NA* | 0·356  (0·324, 0·425) | 4766·2  (4314·6, 5271·6) | *Ref.* | 68·2  (47·6, 86·5) | 68·0  (43·5, 85·6) | 0·750  (0·730, 0·779) | *Ref.* |
| Model 2 ^b^ | 1·10 (1·05, 1·14), p<0·001 | 0·370  (0·338, 0·441) | 4751·9  (4299·8, 5255·4) | -14·3  (-35·1, -4·1) * | 69·7  (45·6, 80·3) | 68·0  (54·4, 88·6) | 0·755  (0·737, 0·785) | 0·006  (0·002, 0·015) * |
| **Women (N cases/ N total = 92/1030)** | | | | | | | | |
| Model 1 ^a^ | *NA* | 0·435  (0·369, 0·583) | 1190·7  (963·9, 1404·2) | *Ref.* | 67·7  (51·2, 88·6) | 78·2  (54·7, 94·6) | 0·763  (0·735, 0·827) | *Ref.* |
| Model 2 ^b^ | 1·12 (1·02, 1·23), p=0·013 | 0·454  (0·387, 0·592) | 1186·5  (957·2, 1399·4) | -4·2  (-25·4, 1·6) | 70·8  (51·8, 90·2) | 76·4  (57·0, 95·6) | 0·774  (0·750, 0·837) | 0·011  (0·001, 0·036) * |

Abbreviations: HR: Hazard ratio; CI: confidence interval; AIC: Akaike information criterion; C-index: Harrell’s C-index; PA, physical activity; SB, sedentary behaviour.

* Confidence interval not containing zero indicates significant difference in predictive performance between Model 1 and Model 2

^a^ Model 1: Model includes socio-demographic, behavioural, and health-related factors

^b^ Model 2: Model 1 additionally adjusted for the physical activity and sedentary behaviour composite score 1

^c^ Youden index cutoff points for the calculation of the sensitivity and specificity are as follows: among men: 0·362 (95% CI: [0·274, 0·477]) for Model 1 and 0·378 (95% CI: [0·290, 0·495]) for Model 2; among women: 0·459 (95% CI: [0·361, 0·629]) for Model 1 and 0·471 (95% CI: [0·386, 0·651]) for Model 2

Supplementary Table S6. Predictive performance of physical activity and sedentary behaviour composite scores for mortality risk by body mass index status in Whitehall II accelerometer sub-study

|  | **HR (95% CI) of**  **the PA and SB**  **composite score 1** | **Royston’s R²**  **(95% CI)** | **AIC**  **(95% CI)** | **ΔAIC**  **(95% CI)** | **Sensitivity %**  **(95% CI) ^c^** | **Specificity %**  **(95% CI) ^c^** | **C-index**  **(95% CI)** | **ΔC-index**  **(95% CI)** |
| --- | --- | --- | --- | --- | --- | --- | --- | --- |
| **Body mass index < 25kg/m² (N cases/ N total = 165/1548)** | | | | | | | | |
| Model 1 ^a^ | *NA* | 0·408  (0·359, 0·510) | 2242·5  (1901·4, 2542·5) | *Ref.* | 74·8  (46·9, 89·6) | 64·7  (48·8, 91·7) | 0·769  (0·741, 0·812) | *Ref.* |
| Model 2 ^b^ | 1·07 (1·01, 1·14), p=0·016 | 0·413  (0·366, 0·516) | 2238·9  (1897·5, 2534·8) | -3·6  (-19·9, 1·4) | 66·4  (45·1, 87·2) | 73·5  (51·5, 93·0) | 0·775  (0·749, 0·817) | 0·006  (0·000, 0·018) * |
| **Body mass index 25-29·9 kg/m² (N cases/ N total = 171/1720)** | | | | | | | | |
| Model 1 ^a^ | *NA* | 0·395  (0·346, 0·493) | 2381·6  (2051·4, 2718·0) | *Ref.* | 58·1  (49·3, 93·6) | 79·4  (43·2, 91·5) | 0·759  (0·737, 0·800) | *Ref.* |
| Model 2 ^b^ | 1·12 (1·05, 1·20), p<0·001 | 0·407  (0·365, 0·510) | 2371·6  (2046·1, 2703·6) | -10·0  (-31·7, -0·8) * | 59·9  (51·8, 86·9) | 83·8  (52·4, 89·4) | 0·767  (0·743, 0·807) | 0·008  (0·001, 0·022) * |
| **Body mass index ≥ 30 kg/m² (N cases/ N total = 74/723)** | | | | | | | | |
| Model 1 ^a^ | *NA* | 0·347  (0·305, 0·531) | 927·6  (726·6, 1109·3) | *Ref.* | 75·2  (33·3, 91·6) | 75·0  (44·7, 100) | 0·748  (0·720, 0·824) | *Ref.* |
| Model 2 ^b^ | 1·11 (1·00, 1·23), p=0·041 | 0·364  (0·325, 0·540) | 925·5  (727·3, 1108·8) | -2·1  (-18·6, 1·7) | 60·2  (35·1, 90·4) | 81·2  (52·6, 100) | 0·755  (0·730, 0·830) | 0·006  (-0·001, 0·032) |

Abbreviations: HR: Hazard ratio; CI: confidence interval; AIC: Akaike information criterion; C-index: Harrell’s C-index; PA, physical activity; SB, sedentary behaviour.

* Confidence interval not containing zero indicates significant difference in predictive performance between Model 1 and Model 2

^a^ Model 1: Model includes socio-demographic, behavioural, and health-related factors

^b^ Model 2: Model 1 additionally adjusted for the physical activity and sedentary behaviour composite score 1

^c^ Youden index cutoff points for the calculation of the sensitivity and specificity are as follows: among those with BMI<25 kg/m²: 0·395 (95% CI: [0·327, 0·571]) for Model 1 and 0·400 (95% CI: [0·345, 0·580]) for Model 2; among those with BMI 25-29·9 kg/m²: 0·375 (95% CI: [0·307, 0·548]) for Model 1 and 0·437 (95% CI: [0·332, 0·597]) for Model 2; among those with BMI : 0·502 (95% CI: [0·307, 0·726]) for Model 1 and 0·414 (95% CI: [0·316, 0·724]) for Model 2

Supplementary Table S7. Predictive performance of physical activity and sedentary behaviour composite scores for mortality risk by morbidity status in Whitehall II accelerometer sub-study

|  | **HR (95% CI) of**  **the PA and SB**  **composite score 1** | **Royston’s R²**  **(95% CI)** | **AIC**  **(95% CI)** | **ΔAIC**  **(95% CI)** | **Sensitivity %**  **(95% CI) ^c^** | **Specificity %**  **(95% CI) ^c^** | **C-index**  **(95% CI)** | **ΔC-index**  **(95% CI)** |
| --- | --- | --- | --- | --- | --- | --- | --- | --- |
| **Participants without chronic disease (N cases/ N total = 182/2362)** | | | | | | | | |
| Model 1 ^a^ | *NA* | 0·349  (0·306, 0·454) | 2667·6  (2285·1, 2990·8) | *Ref.* | 77·0  (44·5, 86·0) | 62·9  (52·8, 91·3) | 0·743  (0·715, 0·786) | *Ref.* |
| Model 2 ^b^ | 1·09 (1·03, 1·16), p=0·006 | 0·366  (0·320, 0·470) | 2662·0  (2284·7, 2993·4) | -5·6  (-25·3, 0·9) | 76·7  (46·0, 85·7) | 62·9  (53·8, 90·8) | 0·746  (0·718, 0·789) | 0·003  (-0·001, 0·015) |
| **Participants with at least one chronic disease (N cases/ N total = 228/1629)** | | | | | | | | |
| Model 1 ^a^ | *NA* | 0·349  (0·309, 0·439) | 3163·5  (2794·7, 3559·5) | *Ref.* | 72·4  (34·3, 84·4) | 63·5  (49·8, 98·3) | 0·743  (0·723, 0·782) | *Ref.* |
| Model 2 ^b^ | 1·11 (1·05, 1·17), p<0·001 | 0·367  (0·332, 0·453) | 3150·3  (2781·5, 3544·1) | -13·2  (-38·0, -2·1) * | 46·5  (36·5, 90·2) | 88·9  (47·4, 98·3) | 0·754  (0·735, 0·789) | 0·011  (0·003, 0·026) * |

Abbreviations: HR: Hazard ratio; CI: confidence interval; AIC: Akaike information criterion; C-index: Harrell’s C-index; PA, physical activity; SB, sedentary behaviour.

* Confidence interval not containing zero indicates significant difference in predictive performance between Model 1 and Model 2

^a^ Model includes socio-demographic, behavioural, and health-related factors

^b^ Model 1 additionally adjusted for physical activity and sedentary behaviour composite score 1

^c^ Youden index cutoff points for the calculation of the sensitivity and specificity are as follows: among those without chronic disease: 0·400 (95% CI: [0·306, 0·539]) for Model 1 and 0·396 (95% CI: [0·314, 0·538]) for Model 2; among those with at least one chronic disease: 0·359 (95% CI: [0·309, 0·537]) for Model 1 and 0·354 (95% CI: [0·312, 0·546]) for Model 2

Supplementary Table S8. Sample characteristics in 2014-2017 by mortality status at the end of follow-up (February 2021) in CoLaus accelerometer sub-study

|  | **All-cause mortality** | | ***p*-value** |
| --- | --- | --- | --- |
|  | **No (N = 1224)** | **Yes (N = 105)** |  |
| **Socio-demographic factors** |  |  |  |
| Age (years), M (SD) | 69·1 (6·2) | 74·8 (7·2) | <0·001 |
| Women | 716 (58·5%) | 37 (35·2%) | <0·001 |
| Non-white ethnicity | 64 (5·2%) | 2 (1·9%) | 0·132 |
| Education |  |  |  |
| Mandatory education | 179 (14·6%) | 16 (15·2%) | 0·23 |
| Apprenticeship | 492 (40·2%) | 49 (46·7%) |  |
| High school | 334 (27·3%) | 19 (18·1%) |  |
| University | 219 (17·9%) | 21 (20·0%) |  |
| Not married/cohabitating | 699 (57·1%) | 55 (52·4%) | 0·35 |
| **Behavioural factors** |  |  |  |
| Smoking status |  |  |  |
| Never smoker | 529 (43·2%) | 30 (28·6%) | 0·006 |
| Past smoker | 524 (42·8%) | 52 (49·5%) |  |
| Current smoker | 171 (14·0%) | 23 (21·9%) |  |
| Alcohol intake |  |  |  |
| 0 unit/week | 294 (24·0%) | 31 (29·5%) | 0·35 |
| 1-14 units/week | 784 (64·1%) | 60 (57·1%) |  |
| > 14 units/week | 146 (11·9%) | 14 (13·3%) |  |
| Fruit and vegetable intake |  |  |  |
| Less than daily | 16 (1·3%) | 6 (5·7%) | <0·001 |
| Daily | 366 (29·9%) | 21 (20·0%) |  |
| Twice daily or more | 842 (68·8%) | 78 (74·3%) |  |
| **Health-related factors** |  |  |  |
| Body mass index |  |  |  |
| Normal (< 25 kg/m²) | 428 (35·0%) | 30 (28·6%) | 0·323 |
| Overweight (25-29·9 kg/m²) | 530 (43·3%) | 47 (44·8%) |  |
| Obesity (≥ 30 kg/m²) | 266 (21·7%) | 28 (26·7%) |  |
| Hypertension | 659 (53·8%) | 73 (69·5%) | 0·002 |
| Hyperlipidemia | 460 (37·6%) | 45 (42·9%) | 0·29 |
| Diabetes | 140 (11·4%) | 30 (28·6%) | <0·001 |
| Number of chronic diseases,^a^ M (SD) | 0·2 (0·5) | 0·6 (0·7) | <0·001 |
| Presence of ADL,^b^ M (SD) | 70 (5·7%) | 10 (9·5%) | 0·12 |
| Presence of IADL,^c^ M (SD) | 93 (7·6%) | 20 (19·0%) | <0·001 |
| **Accelerometer variables** |  |  |  |
| ***Overall activity level****, M (SD)* |  |  |  |
| Mean acceleration (m*g*) | 39·0 (11·5) | 28·5 (10·8) | <0·001 |
| ***Total duration (min)****, M (SD)* |  |  |  |
| Sedentary behaviour | 636·7 (116·7) | 727·2 (142·6) | <0·001 |
| Light intensity physical activity | 247·5 (70·5) | 200·8 (94·2) | <0·001 |
| Moderate-to-vigorous physical activity | 84·1 (53·6) | 38·4 (39·8) | <0·001 |
| ***Bouts duration****, M (SD)* |  |  |  |
| Mean duration (min) of activity bout of |  |  |  |
| Sedentary behaviour | 9·8 (4·3) | 14·7 (13·3) | < 0·001 |
| Light intensity physical activity | 2·5 (0·4) | 2·5 (0·5) | 0·65 |
| Moderate-to-vigorous physical activity | 2·2 (0·6) | 1·7 (0·6) | <0·001 |
| Time (min/day) in < 10 min bouts of |  |  |  |
| Sedentary behaviour | 155·2 (40·4) | 132·2 (51·1) | <0·001 |
| Light intensity physical activity | 213·4 (54·5) | 169·9 (73·3) | <0·001 |
| Moderate-to-vigorous physical activity | 69·4 (39·9) | 34·4 (34·3) | <0·001 |
| Time (min/day) in ≥ 10 min bouts of |  |  |  |
| Light intensity physical activity | 34·1 (24·3) | 30·9 (27·1) | 0·20 |
| Moderate-to-vigorous physical activity | 14·7 (19·0) | 3·9 (8·8) | <0·001 |
| Time (min/day) in 10-29·9 min bouts of |  |  |  |
| Sedentary behaviour | 183·0 (42·6) | 168·1 (51·7) | <0·001 |
| Time (min/day) in ≥ 30 min bouts of |  |  |  |
| Sedentary behaviour | 298·4 (131·0) | 426·9 (184·2) | <0·001 |
| ***Frequency****, M (SD)* |  |  |  |
| Number (N/day) of bouts of |  |  |  |
| Sedentary behaviour | 72·6 (15·2) | 64·2 (20·1) | <0·001 |
| Light intensity physical activity | 97·7 (22·7) | 77·1 (29·4) | <0·001 |
| Moderate-to-vigorous physical activity | 35·8 (17·7) | 19·3 (17·0) | <0·001 |
| Number of days with ≥30 min of moderate-to-vigorous  physical activity | 5·7 (2·0) | 2·9 (3·0) | <0·001 |
| ***Intensity distribution****, M (SD)* |  |  |  |
| Intensity intercept | 12·3 (0·5) | 12·7 (0·7) | <0·001 |
| Intensity gradient | -2·02 (0·18) | -2·22 (0·25) | <0·001 |
| ***Timing****, ^e^ M (SD)* |  |  |  |
| Timing of physical activity (hours) | 7h 12min  (6h 6min) | 7h 48min  (6h 24min) | 0·31 |

Data are N (%), otherwise specified. Abbreviations: M: mean; SD: standard deviation; ADL: activities of daily living; IADL: instrumental activities of daily living.

^a^ Chronic disease include coronary heart disease, stroke, heart failure, and depression.

^b^ Assessed by the question: “The last 4 weeks, did you feel difficult to take care of you?”.

^c^ Assessed by the question: “The last 4 weeks, did you feel difficult to go shopping?”.

^d^ Timing of five most active hours during the waking period.
